# Multidrug-Resistant Bacterial Colonization and Infections in Large Retrospective Cohort of Mechanically Ventilated COVID-19 Patients^1^

**DOI:** 10.3201/eid2908.230115

**Published:** 2023-08

**Authors:** Davide Mangioni, Liliane Chatenoud, Jacopo Colombo, Emanuele Palomba, Fernando A. Guerrero, Matteo Bolis, Nicola Bottino, Giuseppe Breda, Maria V. Chiaruttini, Gabriele Fior, Manuela Marotta, Giovanni Massobrio, Caterina Matinato, Antonio Muscatello, Paola Previtali, Sara Santambrogio, Francesca Tardini, Gianluca Zuglian, Giacomo Grasselli, Roberto Fumagalli, Andrea Gori, Nino Stocchetti, Gianpaola Monti, Alessandra Bandera, the MDR in FIERA Study Group

**Affiliations:** Foundation IRCCS Ca’ Granda Ospedale Maggiore Policlinico di Milano, Milan, Italy (D. Mangioni, E. Palomba, M. Bolis, N. Bottino, G. Breda, G. Massobrio, C. Matinato, A. Muscatello, G. Zuglian, G. Grasselli, A. Gori, N. Stocchetti, A. Bandera);; University of Milan, Milan (D. Mangioni, E. Palomba, G. Grasselli, A. Gori, N. Stocchetti, A, Bandera);; Istituto di Ricerche Farmacologiche Mario Negri IRCCS, Milan (L. Chatenoud, M.V. Chiaruttini);; Harvard Medical School, Boston, Massachusetts, USA (J. Colombo);; ASST Grande Ospedale Metropolitano Niguarda, Milan (J. Colombo, F.A. Guerrero, G. Fior, M. Marotta, P. Previtali, S. Santambrogio, F. Tardini, R. Fumagalli, G. Monti);; ULSS 3 Serenissima, Venice, Italy (G. Zuglian);; University of Milano-Bicocca, Milan (R. Fumagalli)

**Keywords:** antimicrobial resistance, multidrug resistant, MDR, multidrug-resistant bacteria, bacteria, multidrug-resistant organism, MDRO, bacteria, colonization, infections, large retrospective cohort, COVID-19, respiratory infections, mechanically ventilated patients, SARS-CoV-2, intensive care unit, incidence rate, antimicrobial stewardship, zoonoses

## Abstract

Few data are available on incidence of multidrug-resistant organism (MDRO) colonization and infections in mechanically ventilated patients, particularly during the COVID-19 pandemic. We retrospectively evaluated all patients admitted to the COVID-19 intensive care unit (ICU) of Hub Hospital in Milan, Italy, during October 2020‒May 2021. Microbiologic surveillance was standardized with active screening at admission and weekly during ICU stay. Of 435 patients, 88 (20.2%) had MDROs isolated ≤48 h after admission. Of the remaining patients, MDRO colonization was diagnosed in 173 (51.2%), MDRO infections in 95 (28.1%), and non-MDRO infections in 212 (62.7%). Non-MDRO infections occurred earlier than MDRO infections (6 days vs. 10 days; p<0.001). Previous exposure to antimicrobial drugs within the ICU was higher in MDRO patients than in non-MDRO patients (116/197 [58.9%] vs. 18/140 [12.9%]; p<0.001). Our findings might serve as warnings for future respiratory viral pandemics and call for increased measures of antimicrobial stewardship and infection control.

Bacterial superinfections represent a major threat for patients in intensive care units (ICUs), severely affecting clinical course and length of hospital stay. The COVID-19 pandemic caused an unprecedented rate of ICU admissions and drastically changed ICU care itself, in terms of infection control measures and therapeutic usage of steroids and immunomodulating drugs. The percentages of hospital-acquired infections (HAIs) in COVID-19 patients vary widely, ranging from 7% to 13% in nonintensive hospital wards and up to 45% in ICUs (*1*–*3*).

Several studies have assessed the burden of multidrug-resistant organisms (MDROs) in COVID-19 patients admitted to ICUs, reporting heterogeneous results with prevalence ranging from 11% to 50% and incidence rate from 4.5 cases/1,000 patient-days to 30 cases/1,000 patient-days (*4*–*21*). However, studies published so far have relevant limitations, often not clearly discriminating between colonization and infection (*8**,**9**,**11**,**12*), and either including small populations or showing heterogeneity in clinical settings and microbiologic surveillance procedures when describing larger pool of persons, such as in multicentric studies (*18*–*20*).

Our study was conducted to address the need for further evidence on incidence and etiology of MDRO colonization and infections in mechanically ventilated COVID-19 patients. We analyzed clinical and microbiologic data systematically collected in a large ICU in northern Italy.

## Methods

### Study Design and Setting

We conducted a retrospective cohort study on routinely collected data of COVID-19 patients admitted to the Milano Fiera ICU during October 23, 2020‒May 31, 2021. This ICU was a large COVID-19 ICU developed in Milan, Italy, to face the effect of the pandemic. It admitted patients who had SARS-CoV-2 infection requiring mechanical ventilation from different healthcare settings: emergency department, nonintensive hospital wards, and other ICUs. This ICU could accommodate up to 100 patients divided into distinct units (modules) managed by ICU staff from different hospitals. Microbiologic surveillance was standardized and consisted of perineal and nasal swab specimens for MDROs and endotracheal aspirate cultures obtained at ICU admission and then once (perineal and nasal swab specimens) or twice (endotracheal aspirate) a week. All modules referred to the IRCCS Ca’ Granda Ospedale Maggiore Policlinico Foundation for laboratory and microbiologic analyses and for infectious diseases specialist consultation.

### Study Participants and Data Collection

All consecutive patients who had laboratory-confirmed SARS-CoV-2 infection and were admitted to the ICU were considered for inclusion. Exclusion criteria were age <18 years, length of mechanical ventilation <48 h, and lack of comprehensive clinical documentation. We collected demographic, clinical, laboratory, and outcome data from clinical records and microbiologic and therapeutic data from dedicated hospital databases (Appendix). The study was registered by the Milan Area 2 Ethical Committee (#701_2021) and was conducted in accordance with standards of the Helsinki Declaration. Written informed consent was waived because of the retrospective nature of the analysis. The study was retrospectively registered at clinicaltrials.gov on March 24, 2022 (identifier: NCT05293418).

### Microbiologic Data Processing

For each patient, we retrieved bacterial isolates from a microbiology database, which were independently reviewed by dedicated intensivists and infectious disease specialists and classified as contamination, colonization, or infection, according to international guidelines (Appendix) (*22*,*23*). In brief, infections were defined by the presence of a major bacterial load associated with clinical manifestations within the infection window period (±3 days from specimen collection) (*22*,*23*), Isolates were classified as colonization when no adverse clinical signs or symptoms were documented. We defined contamination as all microbiologic isolates that did not meet the criteria of infection or colonization and that were listed in the US Centers for Disease Control and Prevention National Healthcare Safety Network (https://www.cdc.gov/nhsn/index.html) list of common commensals. We retained only the first species-specific MDRO colonization of each patient for further analysis.

We distinguished new infectious episodes from persistent infections according to the European Centre for Disease Prevention and Control definitions (*23*). We stratified infection episodes as infection without sepsis, sepsis or septic shock according to Sepsis-3 criteria (*24*). We defined secondary bloodstream infections (BSIs) by using the secondary BSI attribution period according to the Centers for Diseases Control and Prevention National Healthcare Safety Network (*22*). We also defined isolates as MDROs when they were nonsusceptible to >1 agents in >3 antimicrobial drug categories (*25*) or when harboring specific antimicrobial drug resistance mechanisms (e.g., methicillin-resistant *Staphylococcus* spp., vancomycin-resistant *Enterococcus* spp., extended-spectrum β-lactamase/AmpC/carbapenemases‒producing Enterobacterales) by using rapid detection methods (*4*).

### Statistical Analysis

We reported patient characteristics overall and for selected groups of interest, such as MDROs acquired before/after ICU admittance and MDRO infection/colonization. Medians (interquartile range [IQRs]) are reported for continuous variables and numbers (percentages) for categorical variables. We calculated crude incidence rates (IRs) per 1,000 patient-days and relative 95% CIs, considering for each patient any first species-specific MDRO colonization or each new MDRO/non-MDRO HAI (*26*). We used SAS version 9.4 software (SAS Institute, https://www.sas.com) for statistical analysis (Appendix).

## Results

### Population Description

**Figure 1:**
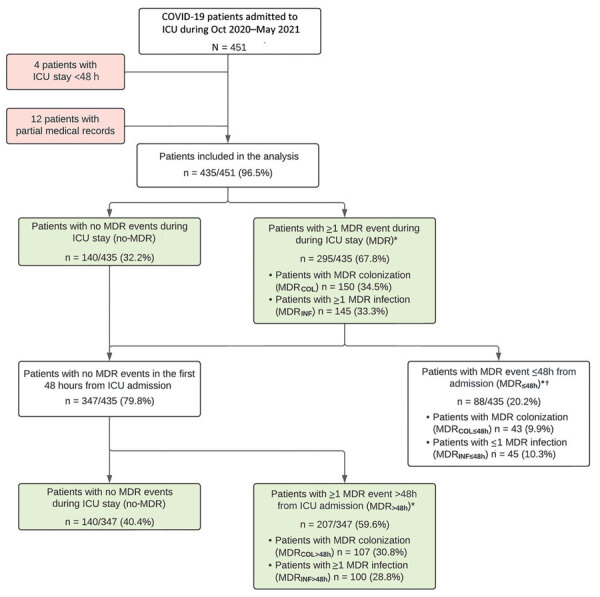
Study flowchart showing patient selection process for multidrug-resistant bacterial colonization and infections in large retrospective cohort of COVID-19 mechanically ventilated patients admitted to ICU in Milan, Italy, October 2020–May 2021. ICU, intensive care unit; MDR, multidrug resistant. Asterisks indicate subgroups. *Patients are grouped on the basis of the worst MDR event diagnosed in MDR colonization or MDR infection, irrespective of the presence of previous or later MDR colonization. †At ICU admission, there were 78 colonizations and 10 infections. During ICU stay, 35/78 (44.9%) colonized patients had MDR infections develop.

A total of 451 patients from 46 different hospitals were admitted to ICUs during October 2020‒May 2021. Of those, 435 were included in the analysis. We provide details of the patient selection process (Figure 1) and trends of patient admission by referring hospital per month (Appendix Figure 1).

Only 12/435 patients (2.7%) were reported to have MDRO colonization/infection before ICU admission. In 88/435 patients (20.2%), MDRO were isolated within 48 h upon entry to the ICU (MDR_≤48h_), and those patients were similarly distributed between referring hospitals (Appendix Figure 2). This group was composed of 78 colonizations and 10 infections; 35/78 (44.9%) colonized patients subsequently had MDRO infections develop. Compared with the 347 patients who had no evidence of MDRO during the first 48 hours of ICU stay (no-MDR+MDR_>48h_), the MDR_≤48h_ group was characterized by higher admittance from other ICUs and lower admittances from emergency departments (ICU 31/88 [35.2%] in MDR_≤48h_ vs. 86/347 [24.8%]) in no-MDR+MDR_>48h_; emergency department 15/88 [17.1%] in MDR_≤48h_ vs. 102/347 [29.4%] in no-MDR+MDR_>48h_). The MDR_≤48h_ group showed slightly longer (although not significantly) length of stay in the ICU of origin than patients who developed MDRO events later during their stay and to no-MDR patients (medians 11.5, 9, and 7 days, respectively; p = 0.09). The MDR_≤48h_ group was also characterized by a larger amount of antimicrobial drug intake before ICU admission (no antimicrobial drug in 25/88 [28%] of MDR_≤48h_ vs. 126/327 [36.3%] of no-MDR+MDR_>48h_; >3 classes of antimicrobial drugs in 12/88 [13.6%] of MDR_≤48h_ vs. 23/347 [6.6%] of no-MDR+MDR_>48h_). We compiled demographic and clinical characteristics by groups (Appendix Table 1) and duration between hospitalization and transfer to the ICU on the basis of patients’ setting of provenance (Appendix Table 2).

Of the 347 patients who had no MDRO isolates within the first 48 hours from ICU admission, 207 (67.5%) had >1 MDRO event (MDR_>48h_); 107 (30.8%) patients had MDRO colonization only (MDR_COL>48h_) and 100 (28.8%) had >1 MDRO infection (MDR_INF>48h_) (Figure 1). We compiled patient characteristics and outcomes (Table 1) overall and for no-MDR and MDR_>48h_ patients, further stratified as MDR_COL>48h_ and MDR_INF>48h_. Median age was 65 years (IQR 59–71 years); 95/347 (27.4%) patients were female. More than 80% of patients had >1 concurrent condition, and hypertension was the most common (181/347, 52.2%). Patients who had ever smoked were more frequent in the MDR_INF>48h_ group (26/100, 26%) than in the MDR_COL>48h_ group (11/107, 10.3%; p = 0.003). Transfer to the ICU occurred mostly from nonintensive hospital wards (159/347, 45.8%), but relevant proportions were transferred directly from the emergency department (102/347, 29.4%) or from other ICUs (86/347, 24.8%). Patients were transferred to ICU early during hospitalization, a median time of 5 days from first hospital admittance. 

**Table 1:** Characteristics of patients admitted to ICU in Milan, Italy, October 2020–May 2021, who had no MDRO isolates within the first 48 hours after admission*

Characteristics	Total, n = 347	No-MDR, n = 140	MDR_>48h_, n = 207	MDR_COL>48h_, n = 107	MDR_INF>48h_, n = 100
Demographics					
Age, y (range)	65.0 (59.0–71.0)	65.5 (59.0–71.0)	65.0 (59.0–71.0)	65.0 (60.0–71.0)	65.0 (58.0–71.0)
Sex					
M	252 (72.6)	102 (72.9)	150 (72.5)	73 (69.2)	77 (77.0)
F	95 (27.4)	38 (27.1)	57 (27.5)	34 (31.8)	23 (23.0)
BMI, kg/m**^2^** (range)	28.0 (26.0–31.0)	28.0 (26.0–31.0)	29.0 (26.0–32.0)	29.0 (26.0–32.0)	29.0 (26.0–32.0)
Obesity, BMI >30	122 (35.2)	45 (32.1)	77 (37.2)	35 (32.7)	42 (42.0)
Ever smoker†	66 (19.0)	29 (20.7)	37 (17.9)	11(10.3)	26 (26.0)
Concurrent conditions					
Hypertension	181 (52.2)	67 (47.9)	114 (55.0)	58 (54.2)	56 (56.0)
Cardiovascular disease	92 (26.6)	38 (27.1)	54 (26.2)	34 (31.8)	20 (20.0)
Pneumopathy	48 (13.8)	20 (14.3)	28 (13.5)	12 (11.2)	16 (16.0)
Neuropathy	14 (4.0)	8 (5.7)	6 (2.9)	4 (3.7)	2 (2.0)
Diabetes	69 (19.9)	32 (22.9)	37 (17.9)	21 (19.6)	16 (16.0)
Immunologic deficits‡	22 (6.3)	9 (6.4)	13 (6.3)	9 (8.4)	4 (4.0)
No. concurrent conditions					
0	66 (19.0)	26 (18.6)	40 (19.3)	22 (20.6)	18 (18.0)
1	115 (33.1)	50 (35.7)	65 (31.4)	30 (28.0)	35 (35.0)
2	87 (25.1)	31 (22.1)	55 (27.0)	27 (25.2)	29 (29.0)
>3	79 (22.3)	33 (23.6)	46 (22.2)	28 (26.2)	18 (18.0)
Setting characteristics					
Month of ICU admission					
Oct 2020	18 (5.2)	6 (4.3)	12 (5.8)	8 (7.5)	4 (4.0)
Nov 2020	76 (21.9)	33 (23.6)	43 (20.8)	19 (17.8)	24 (24.0)
Dec 2020	34 (9.8)	13 (9,3)	21 (10.1)	9 (8.4)	12 (12.0)
Jan 2021	48 (13.8)	14 (10.0)	34 (16.4)	17 (15.9)	17 (17.0)
Feb 2021	41 (11.8)	21 (15.0)	20 (9.7)	14 (13.1)	6 (6.0)
Mar 2021	64 (18.4)	25 (17.9)	39 (18.4)	24 (22.4)	15 (15.0)
Apr 2021	54 (15.6)	21 (15.0)	33 (15.9)	13 (12.2.)	20 (20.0)
May 2021	12 (3.5)	7 (5.0)	5 (2.4)	3 (2.8)	2 (2.0)
Setting of provenance					
ED	102 (29.4)	47 (33.6)	55 (26.6)	32 (29.9)	23 (23.0)
Nonintensive hospital wards	159 (45.8)	67 (47.9)	92 (44.4)	44 (41.1)	48 (48.0)
ICU	86 (24.8)	26 (18.6)	60 (29.0)	31 (29.0)	29 (29.0)
Hospital center					
A	47 (13.5)	16 (11.4)	31 (15.0)	16 (15.0)	15 (15.0)
B	28 (8.1)	12 (8.6)	16 (7.7)	11 (10.3)	5 (5.0)
C	30 (8.7)	11 (7.9)	19 (9.2)	6 (5.6)	13 (13.0)
D	22 (6.3)	4 (2.9)	18 (8.7)	7 (6.5)	11 (11.0)
E	27 (27.8)	10 (7.1)	17 (8.2)	10 (9.4)	7 (7.0)
F	22 (6.3)	8 (5.7)	14 (6.8)	7 (6.5)	7 (7.0)
G	11 (3.2)	4 (2.9)	7 (3.4)	2 (1.9)	5 (5.0)
H	16 (4.6)	6 (4.3)	10 (4.8)	4 (3.7)	6 (6.0)
I	13 (3.6)	7 (5.0)	6 (2.9)	3 (2.8)	3 (3.0)
J	13 (3.8)	6 (4.3)	7 (3.4)	6 (5.6)	1 (1.0)
K	8 (2.3)	4 (2.9)	4 (1.9)	2 (1.9)	2 (2.0)
Other	109 (31.4)	52 (37.1)	57 (27.5)	32 (29.9)	25 (25.0)
Disease characteristics before ICU admission					
Days from first symptoms to hospitalization (range)	5.0 (3.0–7.0)	6.0 (3.0–8.0)	5.0 (3.0–7.0)	9.0 (3.0–7.0)	5.0 (3.0–7.0)
Days from hospitalization to ICU admission (range)	5.0 (2.0–8.0)	4.0 (2.0–8.0)	5.0 (2.0–9.0)	5.0 (2.0–8.0)	5.0 (3.0–9.0)
Days from hospitalization to MV start (range)	3.0 (1.0–6.0)	3.0 (1.0–6.0)	3.0 (1.0–7.0)	5.0 (1.0–6.0)	4.0 (1.0–7.0)
Steroid therapy§	252 (72.6)	98 (70.0)	154 (74.4)	79 (73.8)	75 (75.0)
Standard dose	228 (65.7)	85 (60.8)	143 (69.0)	75 (70.1)	68 (68.0)
High dose	39 (11.3)	21 (15.0)	18 (8.7)	9 (8.4)	9 (9.0)
Antimicrobial drug therapy					
None	126 (36.3)	48 (34.3)	78 (37.7)	45 (42.1)	33 (33.0)
1 class	125 (36.0)	44 (31.4)	81 (39.1)	39 (36.5)	42 )42.0)
2 classes	73 (21.0)	39 (27.9)	34 (16.4)	14 (13.1)	20 (20.0)
>3 classes	23 (6.6)	9 (6.4)	14 (6.8)	9 )8.4)	5 (5.0)
MDRO infection/colonization	4 (1.2)	2 (1.4)	2 (1.0)	2 (1.9)	0
PaO_2_:FIO_2_ ratio at ICU admission, mm Hg (range)	137 (106.0–180.0)	130.5 (100.5–176.5)	138.0 (110.0–180.0)	147.0 (111.0–184.0)	127.5 (109.0–177.5)
200	56 (16.7)	22 (15.7)	36 (17.4)	19 (17.8)	17 (17.0)
<100 and >200	218 (62.8)	83 (59.3)	135 (65.2)	73 (68.2)	62 (62.0)
<100	71 (20.4)	35 (25.0)	36 (17.4)	15 (14.0)	21 (21.0)
Outcome					
Alive at discharge	229 (66.0)	93 (66.4)	136 (65.7)	74 (69.2)	62 (62.0)
Deceased	118 (34.0)	47 (33.6)	71 (34.3)	33 (30.8)	38 (38.0)
Length of MV, d (range)¶#	16.0 (10.0–26.0)	14.0 (8.0–21.0)	18.0 (12.0–29.0)	16.5 (11.0–26.0)	20.0 (12.0–30.0)
ICU stay, d (range)**	21.0 (13.0–33.0)	15.5 (10.0–24.0)	25.0 (16.0–37.0)	20.0 (12.0–30.0)	29.0 (21.0–42.0)

*Values are no. (%) except as indicated. Patients who had MDRO events during ICU stay (MDR>48h) are reported globally and divided between those who had >1 MDRO infection (MDR_INF>48h_) and those with MDRO colonization only (MDR_COL>48h_). Comparisons are made between no-MDR and MDR>48h groups and between MDR_INF>48h_ and MDR_COL>48h_ groups. Categorical variables are expressed as frequency (percentages), continuous variables are expressed as median (interquartile range). BMI, body mass index; ED, emergency department; ICU, intensive care unit; MDRO, multidrug resistant organism. MV, mechanical ventilation; Other, 36 centers that had <10 patients. †MDR_COL>48h_ versus MDR_INF>48h_. p value = 0.003 by χ^2^ test. ‡At least 1 of solid organ transplantation, active neoplastic disease, hematologic disease, rheumatologic disease, AIDS, asplenia, chemotherapy in the past 3 mo of neutropenia (n<50 cells/μL), use of biologics, use of corticosteroids (>10 mg/day prednisone or equivalent >3 mo prehospitalization), other forms of immunosuppression (including congenital/genetic forms). §Standard dose in case of use of dexamethasone or methylprednisolone <1 mg/kg/d, high dose in case of use of methylprednisolone >1 mg/kg/d or equivalent (patients could have received both standard and high dose of steroid). ¶No-MDR versus MDR_>48h_. p = 0.001 by Mann–Whitney U test. **#**MDR_COL>48h_ versus MDR_INF>48h_. , p = 0.001 by Mann–Whitney U test. **No-MDR versus MDR_>48h_. p = 0.001 by Mann–Whitney U test.

Groups did not differ for steroid use or antimicrobial drug therapies received before ICU admission. According to clinical practice, steroids had been administered for SARS-CoV-2 infection management in 252/347 (72.6%) patients, mostly (228/347, 65.7%) with only a standard dose (dexamethasone 6 mg/d). Most patients (221/347, 63.7%) had previously received antimicrobial drugs before ICU admission. MDRO events before ICU admission were reported in only 4 patients (1.2%). During ICU stay, 118 patients (34%) died, but there were no significant differences between groups. When compared with no-MDR patients, we found that MDR_>48h_ patients had a longer duration of mechanical ventilation (median 18 vs. 14 days; p = 0.001) and of ICU stay (median 25 vs. 15.5 days; p = 0.001). Those differences were largely caused by the MDR_INF>48h_ group (Table 1).

### Bacterial Isolate Description and Incidence

**Figure 2:**
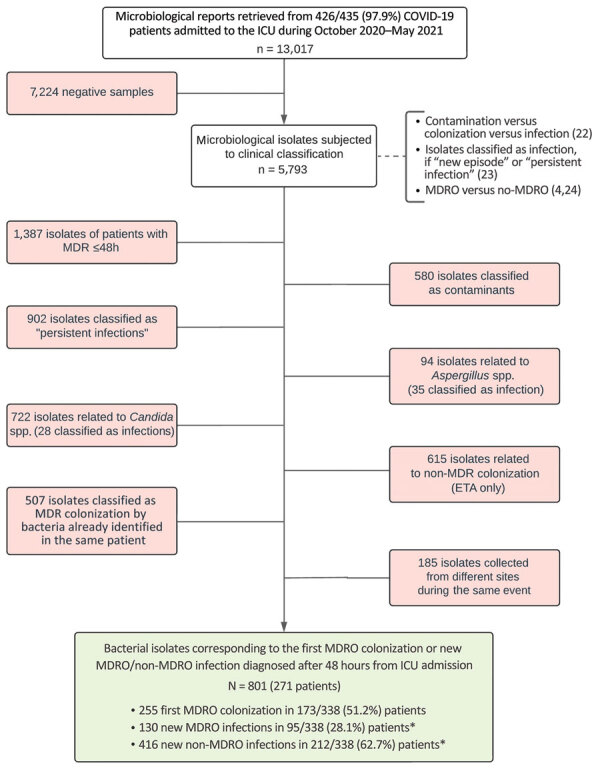
Study flowchart showing microbial isolates selection process for multidrug-resistant bacterial colonization and infections in large retrospective cohort of COVID-19 mechanically ventilated patients admitted to ICU in Milan, Italy, October 2020–May 2021. ETA, emergency treatment area; ICU, intensive care unit; MDR, multidrug resistant; MDRO, multidrug-resistant organism. *Of 338 patients, 159 (47.0%) had either MDRO or non-MDRO infections; 74/338 (21.9%) had both MDRO and non-MDRO infections.

Complete microbiologic reports were available for 426/435 patients, including 338/347 patients (97.4%) with no MDRO isolates within the first 48 hours of ICU admission. We describe the selection process conducted to assess incidences of HAIs and of MDRO events distinguishing between colonization and infection (Figure 2). We identified 801 bacterial isolates from 271 patients that correspond to first MDRO colonization (255 isolates in 173/338 patients, 51.2%) and new episodes of bacterial superinfections, either by MDRO (130 isolates in 95/338 patients, 28.1%) or antimicrobial drug‒susceptible bacteria (non-MDRO, 416 isolates in 212/338 patients, 62.7%). A total of 73 (21.6%) patients had both MDRO colonization and MDRO infection develop during ICU stay, and infections were caused by the same colonizing bacterial species in nearly one third of them (24/73, 32.9%) (Appendix Table 3). Clinical interpretation of bacterial isolates as colonization/infection by attending physicians at the time of arrival of microbiologic results was found to be highly concordant with the retrospective evaluation conducted according to international guidelines (κ coefficient 0.902, 95% CI 0.890–0.913) (Appendix Table 4).

Overall, 546 bacterial HAIs were recorded, 130 (23.8%) caused by MDRO. Gram-negative bacteria accounted for 59.7% (326/546) of all HAIs and for 60% (78/130) of infections caused by MDROs. Bacterial species responsible for HAIs varied by infection site and severity of infection (Appendix Tables 5, 6). Ventilator-associated lower respiratory tract infections (VALRTIs) represented most infectious episodes (359/546, 65.7%), followed by BSI (141, 25.8%) and urinary tract infections (40, 7.3%). Among BSIs, 31/141 (22%) were associated with a central line, 43 (30.5%) were secondary to VALRTI or urinary tract infections, and the remaining 67 (47.5%) were classified as primary BSI without a known bacteremic focus (Appendix Figure 3).

Among MDRO colonization, *Enterococcus faecium* (112/255 isolates, 43.9%) was the most frequent isolate, followed by *Klebsiella* spp. (34, 13.3%), *Escherichia coli* (26, 10.2%), *Staphylococcus aureus* (25, 9.8%), *Pseudomonas aeruginosa* (15, 5.9%) and *Acinetobacter baumannii* (13, 5.1%). We compiled the percentages of MDRO colonization, MDRO HAIs, and non-MDRO HAIs for the most frequently isolated bacteria of the World Health Organization priority pathogens list (*27*) (Appendix Figure 4).

**Figure 3:**
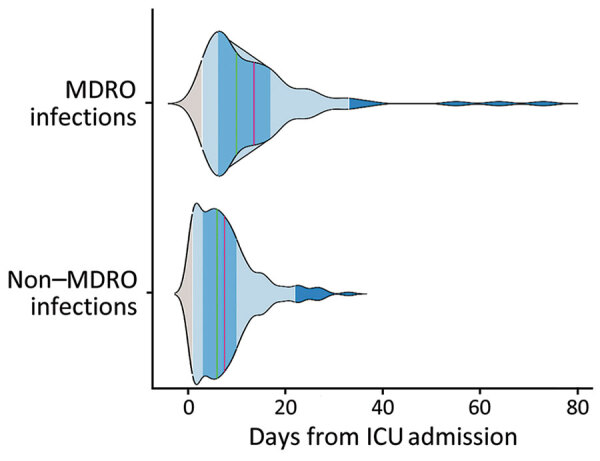
Multidrug-resistant bacterial colonization and infections in large retrospective cohort of COVID-19 mechanically ventilated patients admitted to ICU in Milan, Italy, October 2020–May 2021. Kernel density plot (violin plot) shows healthcare-associated infections by onset time comparing MDRO with non-MDRO. Red lines indicate mean and green lines median onset times; medium blue shading indicates interquartile ranges, and the light blue shading indicates 95% CIs of the mean (p<0.001 by Wilcoxon rank-sum test). ICU, intensive care unit; MDRO, multidrug-resistant organism.

First MDRO colonization occurred at a median time of 13 (IQR 8–12) days after ICU admission. HAIs caused by antimicrobial drug‒susceptible bacteria occurred earlier than in those caused by MDROs at 6 (IQR 3–10) and 10 (IQR 6–17) days from admission (p<0.001) (Figure 3). The incidence rates for MDRO colonization was 29.97 cases/1,000 patient-days (95% CI 26.34–34.10), for MDRO infection was 14.99 cases/1,000 patient-days (95% CI 12.36–18.19), and for non-MDRO infection, was 50.12 cases/1,000 patient-days (95% CI 44.59–56.32). Infection rates varied substantially by infection site (Table 2).

**Table 2:** Incidence rate of MDRO events, overall and divided by infection site, of patients admitted to ICU in Milan, Italy, October 2020–May 2021, who had no MDRO isolates within the first 48 h of admission*

Characteristic	Infections			
	VALRTIs	BSIs	UTIs	Total
MDRO events, first colonization plus new infections	NA	NA	NA	41.68 (36.98–46.99)
First MDRO colonization	NA	NA	NA	29.97 (26.34–34.1)
New MDRO infection	9.44 (7.58–11.74)	4.89 (3.55–6.75)	0.47 (0.14–1.08)	14.99 (12.36–18.19)
New non-MDRO infection	33.25 (29.04–38.07)	11.62 (9.23–14.64)	4.19 (2.97–5.72)	50.12 (44.59–56.32)
Overall new infections, MDRO plus non-MDRO	42.41 (37.81–47.58)	16.57 (13.51–20.31)	5.15 (3.36–6.26)	65.13 (58.76–72.2)

*Values are IR/1,000 person-days (95% CIs). The time considered for IRs was set from ICU admission to discharge, except for VALRTI, where total intubation time was considered. BSIs, bloodstream infections; ICU, intensive care unit; IR, incidence rate; MDRO, multidrug-resistant organism; NA, not applicable (MDRO colonization refers to patients and not infection sites); UTIs, urinary tract infections; VALRTIs, ventilator-associated lower respiratory tract infections.

### Association of Antimicrobial Drugs and Steroids to MDRO Events

We investigated possible associations between MDRO events and previous steroid and antimicrobial drug therapies (Appendix Tables 7, 8). Because steroids were included in the management of COVID-19 pneumonia from the early stage of the disease, we evaluated their intake before and during ICU stay. Almost the entire population had received steroid therapy (313/338, 92.6%), without major differences between no-MDR (132/140, 94.3%), MDR_COL>48h_ (98/103, 95.1%) and MDR_INF>48h_ (83/95, 87.4%) (Appendix Table 7).

To assess possible association between MDRO events and previous antimicrobial drug use, we focused on therapies administered during the first 10 days of ICU stay. This timeline was set to balance observation time between no-MDR and MDR_>48h_ groups because three fourths of MDRO events occurred within this timeframe. Also, three fourths of patients in no-MDR group stayed in ICU >10 days (Appendix Table 8). Previous exposure to antimicrobial drugs was notably higher in patients who developed MDRO events than in patients who did not (116/197 [58.9%] in MDR_>48h_ vs 18/140 [12.9%] in no-MDR; p<0.001) (Appendix Table 8).

## Discussion

We describe incidences and clinical characteristics of HAIs and MDRO events, distinguishing between colonization and infection, in a large cohort of ICU COVID-19 patients from a country with high prevalence of MDRO (*28*). Despite being composed of patients admitted from >45 different hospitals, our cohort is homogeneous for concurrent conditions and risk factors for MDRO acquisition, clinical severity of COVID-19, management of antimicrobial drug therapy, and infection prevention and control strategies within the ICU, including surveillance sampling.

Antimicrobial drug resistance represents a major challenge in the ICU. Its occurrence is the result of the influx of previously colonized patients and acquisition of MDROs during ICU stay, as a consequence of antimicrobial drug overexposure and interpatient transmission, as well as contact with colonized healthcare workers, fomites, or the environment. The incidence of MDROs is strongly influenced by pandemic periods, such as during COVID-19, when unprecedented patient loads in ICUs resulted in breaches in IPC, such as gaps in microbiologic surveillance, lack of communication between clinicians, and reduced attention to environmental measures and contact precautions among healthcare workers (*29*). In addition, ICU admissions caused by viral pandemics place a strain on ICU resources, requiring the reallocation of non-ICU beds, along with the use of non-ICU staff to meet the urgent demand. In this setting, strengthening measures, such as active surveillance with prompt recognition of outbreaks, staff training, increased environmental disinfection and cohorting, become essential to reducing MDRO circulation (*30*).

In the pre–COVID-19 pandemic era, the prevalence of infections caused by MDROs in ICU patients varied from a reported rate of 14.1% in VALRTIs acquired in ICUs in North America (*31*) to an average of >40% in 2 large multicentric worldwide studies of nosocomial BSIs (*32**,**33*). Variability exists between participating countries, ranging from 8% (Australia) to >75%–80% in Asia, eastern Europe, and southern Europe. Carbapenem resistance was present in more than one third of gram-negative bacteria, and 36% of all gram-positive bacteria were MDR (*32**,**33*).

Several studies have been published on MDRO incidence, etiology and source of HAIs in ICU COVID-19 patients (*4*–*21*) (Table 3). Most of those studies evaluated overall MDRO infections or specific HAIs, such as BSI or VALRTIs (*7*,*15*–*17*,*19*,*21*), whereas colonization events were assessed in only a few studies (*8*–*12*,*14*). Incidence measures of MDRO events varied widely; cumulative incidence of the first MDRO event was 5%–57% (*7*,*17*) and incidence rate 2.6–31.48 cases/1,000 patient-days (*11*,*16*). The percentage of MDRO was 27%–100% for all recorded events (*15*,*17*). Compared with the amount of literature evaluating MDRO events during ICU stay, we found that few data are available on MDRO proportions among COVID-19 patients at ICU admission. In recent work of the multicenter HAI-ICU surveillance network in France, the percentage of MDR gram-negative bacteria among >4,000 COVID-19 patients admitted was 11.7% (*34*).

**Table 3:** Characteristics of 18 studies on MDRO colonization and infections in critically ill patients who had COVID-19, Italy*

Year (ref)	Study design		Country		Period		No. ICU COVID-19 patients		MV patients, no. (%)		Median age, y (Q1‒Q3)		Type of MDRO event		MDRO events proportion and measures of incidence		Median days to MDRO event from ICU admission (Q1‒Q3)		Etiology of MDRO colonization/infection†		Source of MDRO infection/colonization		Notes	
2021 (*4*)	Multicentric retrospective study		Italy		2020 Feb‒2020 May		774		688 (89)		62 (54–68)		Infection		Pr MDRO: 272/723 (38%) infections; CI: N/A (359 patients with infections); IR: NA		14 (7–23)		GPB 138/272 (51%); 138/317 (43.5%) within species; *Staphylococcus aureus* 83/272 (31%); 83/151 (55%) within species; *Enterococcus* spp. 29/272 (11%); 29/117 (24.8%) within species; CoNS 24/272 (9%); 24/30 (80%) within species; GNB 133/272 (49%); 133/406 (32.7%) within species *Pseudomonas aeruginosa* 34/272 (12%); 34/119 (28.6%) within species; Enterobacterales (other) 29/272 (11%); 29/101 (28.7%) within species; *Klebsiella* spp. 25/272 (9%); 25/63 (39.7%) within species; *Escherichia coli* 18/272 (7%); 18/54 (33.3%) within species; *Acinetobacter baumannii* 19/272 (7%); 19/21 (90.5%) within species		NA			
2021 (*5*)		Monocentric retrospective study		Spain		3/2020‒6/2020		213		184 (86)		61 (54–68)		Infection		Pr MDRO: 51/174 (29%) infections; CI: 37/213 (17%) patients; IR: NA		28 (13–49)		GPB 10/51 (19.6%); *S. aureus* 3/51 (5.9%); *Enterococcus faecium* 2/51 (3.9%); CoNS 5/51 (9.8%); GNB 41/51 (80.4%); 41/65 (63.5%) within species; *Pseudomonas aeruginosa* 11/51 (21.6%); 11/22 (50%) within species; *Klebsiella* spp. 10/10 (100%) within species		NA			
2021 (*6*)		Monocentric retrospective study		Italy		3/2020‒5/2020		32		NA		68 (55.2–75)		Infection		Pr MDRO: NA; CI: 16/32 (50%) patients; IR: NA		8		*Staphylococcus aureus* 1/23 (4%); *Enterococcus faecium* 2/23 (9%); *Enterococcus faecalis* 1/23 (4%); *Stenotrophomonas maltophilia* 2/23 (9%); *Enterobacter* spp. 2/23 (9%); *Pseudomonas aeruginosa* 3/23 (14%); *Klebsiella pneumoniae* 8/23 (32%); *Acinetobacter baumannii* 4/23 (19%)		Respiratory 2/16 (12.5%); respiratory plus blood 8/16 (50%); blood 2/16 (12.5%); respiratory, urine, and blood 4/16 (25%)			
2021 (*7*)		Monocentric retrospective study		India		7/2020‒12/2021		750		516 (69)		62 (51–72)		Infection (BSI only)		Pr MDRO: NA; CI: 37/750 (5%) patients; IR: NA		NA		*Acinetobacter baumannii* 3/21 (33%) within species; *Klebsiella pneumoniae* 11/14 (78.6%) within species; *Escherichia col*i 6/7 (85.7%) within species; *Pseudomonas aeruginosa* 0/4 (0%) within species		Blood 100%, due to MDRO in 37/64 (57.8%)			
2021 (*8*)		Multicentric retrospective study		Qatar		3/2020‒8/2020		234		NA		49 (40–60)		Infection and colonization (GNB only)		Pr MDRO: NA; CI: 78/234 (33%) patients; IR: 4.5/1,000 pt-days		9 (4–14)		Infection: *tenotrophomonas maltophilia* 24/98 (24.5%); *Klebsiella pneumoniae* 23/98 (23.5%); *Enterobacter cloacae* 18/98 (18.4%); *Escherichia coli* 12/98 (12%); *Serratia marcescens* 12/98 (12%); *Pseudomonas aeruginosa* 8/98 (7.7%); Colonization: *Stenotrophomonas maltophilia* 24/98 (24.5%); *Klebsiella pneumoniae* 23/98 (23.5%); *Enterobacter cloacae* 18/98 (18.4%); *Escherichia coli* 12/98 (12%); *Serratia marcescens* 12/98 (12%); *Pseudomonas aeruginosa* 8/98 (7.7%)		Respiratory 74/98 (75.5%); blood 18/98 (18.4%); urine 6/98 (6.1%)		No distinction between colonization and infection	
2020 (*9*)		Monocentric retrospective study		Belgium		3/2020‒4/2020		75		52 (72)		61 (9) [mean (SD)]		Infection and colonization		Pr MDRO: NA; CI: 24/72 (33%) patients; IR: 30/1,000 pt-days		12 (8–18)		*Enterococcus faecium* 3/31 (10%); *Enterobacter* spp. 13/31 (42%) *Klebsiella pneumoniae* 8/31 (26%); *Escherichia coli* 3/31 (10%); *Pseudomonas aeruginosa* 4/31 (13%)		NA		No distinction between colonization and infection; Mechanisms of resistance (Enterobacterales): AmpC 12/31 (39%); ESBL 9/31 (29%); CRE 3/31 (10%)	
2021 (*10*)		Monocentric retrospective study		Spain		3/2020‒5/2020		24‡		NA		69.5 (45–72)		Colonization		Pr MDRO: NA; CI: 5/24 (21%) patients; IR: NA		NA		*Klebsiella pneumoniae* 3/8 (37.5%); *Escherichia coli* 2/8 (25%); *Stenotrophomonas maltophilia* 1/8 (12.5%); Enterobacterales (other) 2/8 (25%)		NA		4/24 (17%) patients already colonized at ICU admission	
2022 (*11*)		Before-and-after, cross-sectional retrospective study		Italy		1/2020‒4/2020		1,151§		NA		65 (54–74)		Infection and colonization (CPE and CR-Ab only)		CPE colonization; Pr MDRO: NA; CI: NA; IR: 4.02/1,000 pt-days (0.56/,1000 pt-days in MV subset-125 patients); CPE Pr infection; MDRO: NA; NA; IR: 2.6/1000 pt-days (0.72/1000 pt-days in MV subset); CR-Ab colonization; Pr MDRO: NA; CI: N/; IR: 3.68/1,000 pt-days (1.12/1,000 pt-days in MV subset; CR-Ab infection; Pr MDRO: NA; CI NA; IR: 2.6/1,000 pt-days (0.72/1,000 pt-days in MV subset)		CR-Ab; events: 17 (11–24)		NA		CPE colonization: - rectal 32/35 (91.4%); respiratory 3/35 (8.5%); CPE infection: blood 1/2 (50%); respiratory 1/2 (50%); CR-Ab colonization: rectal 5/32 (15%); respiratory 16/32 (50%); CR-Ab infection: blood 9/23 (39%); respiratory 14/23 (60,8%)		Only resistance mechanisms were reported (no bacterial species within Enterobacterales). No distinction between colonization and infection in etiology; Mechanisms of resistance: KPC 22/35 (62.8%); OXA-48 6/35 (17.1%)’ VIM 7/35 (20%); NDM 0/35 (0%)	
2021 (*12*)		Monocentric retrospective study		Italy		2/2021‒5/2021		89		89 (100)		67 (42–83)		Infection and colonization		Pr MDRO: 80/164 (49%) events; CI colonization: 11/89 (12%) patients; IR: NA		14.9 (7–21)		Infection: GPB 7/40 (17.5%); 7/22 (31.8%) within species; *Staphylococcus aureus* 4/40 (10%); 4/12 (33.3%) within species; *Enterococcus faecalis* 2/40 (5%); 2/6 (33.3%) within species; *Enterococcus faecium* 1/40 (2.5%); 1/2 (50%) within species; GNB 33/40 (82.5%); 33/45 (73.3%) within species; *Acinetobacter baumannii* 12/40 (30%); 12/12 (100%) within species; *Pseudomonas aeruginosa* 5/40 (12.5%); 2/4 (50%) within species; *Escherichia coli* 2/40 (5%); *Klebsiella* spp. 6/40 (15%); 6/9 (66.7%) within species; Enterobacterales (other) 8/40 (20%); 8/15 (53.3%) within species; Colonization: GPB 12/37 (32.4%); 12/37 (%) within species; *Staphylococcus aureus* 4/37 (10.8%); 4/17 (23.5%) within species; *Enterococcus faecalis* 4/37 (10.8%); 4/15 (26.6%) within species; *Enterococcus faecium* 4/37 (10.8%); 4/5 (80%) within species; GNB 25/37 (67.6%); 25/60 (41.7%) within species; *Acinetobacter baumannii* 4/37 (10.8%); 4/4 (100%) within species; *Pseudomonas aeruginosa* 4/37 (10.8%); 4/4 (100%) within species; *Escherichia coli* 7/37 (18.9%); 7/25 (28%) within species; *Klebsiella* spp. 2/37 (5.4%); 2/9 (22.2%) within species; Enterobacterales (other) 8/37 (21.6%); 8/18 (44.4%) within species		Respiratory 48/92 (52.2%), due to MDRO in 31/48 (64.5%); blood in 29/92 (31.5%), due to MDRO in 18/31 (62%); urinary in 15/92 (16.3%), due to MDRO in 5/15 (33.3%)		No distinction between colonization and infection in etiology and source	
2022 (*13*)		Monocentric retrospective study		Iran		3/2020‒9/2020		553		337 (61)		69 (21–95)		Infection		Pr MDRO: NA; CI: 62/553 (11%) patients; IR: NA		NA		*Klebsiella pneumoniae* 46/83 (55.4%); 46/47 (97.9%) within species; *Acinetobacter baumannii* 35/83 (42.2%); 35/35 (100%) within species		Respiratory 62/70 (88.6%) blood in 6/70 (8.6%)			
2022 (*14*)		Monocentric prospective study		Thailand		4/2020‒8/2020		120		48 (40)		61 (26–87)		Infection and colonization		Pr MDRO: NA; CI: 28/120 (23%) patients; IR: NA		NA		Infection: *Acinetobacter baumannii* 21/29 (72.4%); *Pseudomonas aeruginosa* 2/29 (6.9%); *Klebsiella pneumoniae* 3/29 (10.3%); *Escherichia coli* 2/20 (6.9%); Colonization: *Acinetobacter baumannii* 21/29 (72.4%); *Pseudomonas aeruginosa* 2/29 (6.9%); *Klebsiella pneumoniae* 3/29 (10.3%); *Escherichia coli* 2/20 (6.9%)		NA		No distinction between colonization and infection	
2021 (*15*)		Monocentric retrospective study		Italy		2/2020‒4/2020		89		82 (93)		61.5 (53.1–68.7)		Infection (BSI only)		Pr MDRO: 32/117 (27%) infections; CI: NA; IR: NA		NA		GPB 10/32 (31.2%); 10/60 (16.7%) within species; *Staphylococcus aureus* 5/32 (15.6%); 5/7 (71.4%) within species; *Enterococcus* spp. 5/32 (15.6%); 5/53 (9.4%) within species; GNB 22/32 (68.8%); 22/29 (75.7%) within species; *Enterobacter* spp. 4/32 (12.5%); 4/6 (66.7%) within species; Enterobacterales (other) 16/32 (50%); 16/19 (84.2%) within species; *Pseudomonas aeruginosa* 1/32 (3.1%); 1/2 (50%) within species; *Stenotrophomonas maltophilia* 1/32 (3.1%); 1/1 (100%) within species		Blood 100%, due to MDRO in 32/85 (37.6%)			
2022 (*16)*		Multicentric retrospective study		Italy		2/2020‒12/2020		316		316 (100)		62 (54–68)		Infection (LRTIs only)		Pr MDRO: 41/144 (28%) infections; CI: 41/316 (13%) patients; IR patients with DEX treatment: 31.48/1,000 pt-days; IR patients without DEX treatment: 27.83/1,000 pt-days		NA		GPB 11/41 (27%); 11/48 (22.9%) within species; *Staphylococcus aureus* 11/41 (27%); 11/40 (27.5%) within species; GNB 30/41 (73%); 30/96 (31.2%) within species; *Pseudomonas aeruginosa* 12/41 (29%); 12/36 (33.3%) within species; *Acinetobacter baumannii* 6/41 (15%); 6/6 (100%) within species; *Escherichia coli* 2/41 (5%); 2/7 (28.6%) within species; *Klebsiella pneumoniae* 1/41 (2.5%); 1/17 (5.9%) within species; Enterobacterales (others) 6/41 (15%); 6/19 (31.6%) within species		LRTI 100%, due to MDRO in 41/144 (28%)			
2022 (*17*)		Monocentric retrospective study		Greece		4/2020‒12/2020		84		84 (100)		70 (57–76)		Infection(BSI only)		Pr MDRO: 60/60 (100%) infections; CI: 48/84 (57%) patients; IR: NA		9 (5–11)		*Acinetobacter baumannii* 20/60 (33%); *Klebsiella pneumoniae* 19/60 (32%); *Enterococcu*s spp. 14/60 (23%); *Stenotrophomonas maltophilia* 3/60 (5%); *Pseudomonas aeruginosa* 2/60 (3%);*Proteus mirabilis* 1/60 (2%); *Serratia marcescens* 1/60 (2%)		BSI 100%, due to MDRO in 60/60 (100%)			
2022 (*18*)		Multicentric retrospective study		Italy		3/2020‒12/2020		123		68 (55.3)		66 (59–75)		Infection (CRE only)		Pr MDRO: NA; CI CRE rectal colonization: 80/106 (75%) patients; IR: NA		16 (10–26)		*Klebsiella pneumoniae* 109/123 (88.6%); *Enterobacter* spp. 10/123 (8.1%); *Proteus mirabilis* 3/123 (2.4%); *Serratia marcescens* 1/123 (0.8%)		Respiratory 28/123 (22.8%); blood 64/123 (52%); urinary 28/123 (22.8%)		Mechanisms of resistance: KPC 63/123 (51.2%); OXA-48 6/123 (4.9%); VIM 7/123 (5.7%); NDM 47/123 (38.2%)	
2022 (*19*)		Multicentric retrospective study		USA		3/2020‒6/2020		148		148 (100)		60 [mean]		Infection (LRTIs only)		Pr MDRO: 20/50 (40%) infections; CI: 8/132 (6%) patients; IR: NA		NA		GPB 6/20 (30%); 6/17 (35.3%) within species; *Staphylococcus aureus* 6/20 (30%); 6/14 (42.8%) within species; GNB 14/20 (70%); 14/33 (%) within species; *Klebsiella* spp. 2/20 (10%); 2/10 (20%) within species; *Pseudomonas* spp. 8/20 (40%); 8/8 (100%) within species; *Escherichia coli* 1/20 (5%); 1/4 (25%) within species; *Stenotrophomonas maltophilia* 2/20 (10%); 2/2 (100%) within species; *Serratia* spp. 1/20 (5%); 1/2 (50%) within species		Respiratory 100%, due to MDRO in 20/50 (40%)			
2022 (*20*)		Two-center retrospective study		Turkey		3/2020‒12/2020		146		116 (79.5)		64 (56–72.3)		Infection (CR GNB only)		Pr MDRO: 61/104 (59%) infections; CI: 41/146 (28%) patients; IR: NA		14		*Acinetobacter* spp. 26/61 (42.6%); 26/27 (96.3%) within species; *Klebsiella* spp. 24/61 (39.3%); 24/26 (92.3%) within species; *Pseudomonas aeruginosa* 7/61 (11.5%); 7/9 (77.8%) within species		NA			
2022 (*21*)		Monocentric retrospective study		India		8/2020‒12/2020		63		63 (100)		62 (12) [mean (SD)]		Infection (LRTIs only)		Pr MDRO: 52/100 (52%) infections; CI: NA; IR: NA		NA		*Klebsiella pneumoniae* 31/52 (56%); *Acinetobacter baumannii* 8/52 (14%); *Burkholderia cepacia* 4/52 (7%); *Enterobacter cloacae* 3/52 (5%); *Pseudomonas aeruginosa* 1/52 (1.8%); *Staphylococcus aureus* 1/52 (1.8%)		Respiratory 100%, due to MDRO in 52/55 (94.5%)			

*BSI, bloodstream infection; CI, cumulative incidence; CoNS, coagulase-negative *Staphylococcus*; CPE, carbapenemases-producing Enterobacterales; CRE, carbapenem-resistant Enterobacterales; CR-Ab, carbapenem-resistant *Acinetobacter baumannii*; CR GNB carbapenem-resistant gram-negative bacteria; DEX, dexamethasone; GNB, gram-negative bacteria; GPB, gram-positive bacteria; IR, incidence rate; LRTI, lower respiratory tract infection; MDRO, multidrug-resistant organism; MV, mechanical ventilation; NA, not available; Pr, proportion; pt-days, patient-days*.*†MDRO proportion by species (no./total MDRO) and, when available, MDRO proportion within species (no./total species) is reported. ‡Subgroup of the study population of 345 COVID-19 patients and no-COVID patients. **§Subgroup of the study population of 2,403 COVID-19 and no-COVID patients.**

In our cohort, 20% of patients had MDRO isolation within the first 48 hours, indicating acquisition before ICU admittance. We found that patients who had MDROs isolated during the first 48 hours were more frequently transferred from other ICUs and exposed to a higher number of antimicrobial drugs before ICU admission. Both of those factors are well known to be associated with development of infections by antimicrobial drug‒resistant pathogens (*6*). Only 2.7% of our cohort had MDRO colonization/infection before ICU admission. The marked difference between expected and observed MDRO prevalence at ICU admission probably reflects the major issues in IPC during the emergency situation of the pandemic mentioned beforehand.

Considering patients without MDRO isolation within the first 48 hours, we observed no differences in demographic characteristics or in clinical severity at admission between patients who showed or not showed development of MDRO events during ICU stay, underlying consistency between groups at ICU admission. In our cohort, we did not find direct association between MDRO infection and in-ICU deaths. However, length of ICU stay and duration of mechanical ventilation were longer for patients with MDRO events and, among them, longer for patients who had infections than for colonized patients. No causative effect can be drawn from these results because occurrence of MDRO events could be either responsible for longer ICU stay or its direct consequence because of longer exposure time (*35*,*36*).

Active surveillance screening coupled with the evaluation of all microbial isolates enabled us to precisely identify patients who had with MDRO events. Two thirds of the cohort showed development of MDRO colonization or infection during ICU stay. Half of our patients were given diagnoses of MDRO colonization during ICU stay, compared with 21% observed in a recent study analyzing a smaller population (*10*). Our results can be, in part, explained by strict routine microbiologic surveillance, which enabled prompt and precise recognition of such cases. Data from previous studies on bacterial superinfections in COVID-19 ICU patients are heterogenous and describe MDRO HAIs in 11%–50% of the population (*6*,*13*). Our results confirm the substantial risk for mechanically ventilated COVID-19 patients to have MDRO infections develop; such infections affected almost 30% of our cohort during ICU stays. Also, more than twice as many patients had antimicrobial drug‒susceptible HAIs.

We found high concordance between clinical diagnosis and retrospective evaluation of HAIs according to literature criteria. We believe this result well demonstrates how implementation of structured antimicrobial stewardship and IPC measures, with collaboration of infectious disease consultants and intensivists, can strongly effect management of critically ill patients, favoring accurate diagnosis and therapeutic choices, according to international guidelines.

Patients who had MDRO events had greater exposure to antimicrobial drugs the first 10 days of ICU stay than patients who had no MDRO findings. This observation is consistent with results of recent studies conducted on large population of patients, which showed major associations between exposure to specific antimicrobial drug classes and drug resistance, and a decreasing pattern over time (*37*,*38*). However, accurate analysis of the association between antimicrobial drug exposure and MDRO events was beyond the scope of this study because other variables, such as average intake time of each antimicrobial drug class and infections with antimicrobial drug‒susceptible bacteria during the observation time, should be considered.

The first limitation of this study is that it was a retrospective monocentric cohort and, therefore, had intrinsic risks of limited accuracy and generalizability. However, interpretation of all microbiologic findings has been conducted ex post on the basis of standardized literature criteria and independent from the physicians’ view. Also, even though the study was monocentric, patients were admitted from >45 hospitals and assisted by different hospital staff. Advantages to this study design derive from the standardized microbiologic surveillance, both in terms of timing and laboratory method, as well as from homogeneous antimicrobial stewardship and IPC strategies among ICU modules. This factor enabled us to provide precise and consistent data in terms of incidence of HAIs and MDRO events, not only infections but also colonization.

Second, this study was not conducted for evaluation of the effect of antimicrobial drugs on development of MDRO or the effect of MDRO events on ICU deaths and length of stay; the sample size was probably inadequate for these issues. Therefore, our findings on this issue should be interpreted with caution.

Third, patients’ data before ICU admission were retrieved from information registered at ICU entry and not from hospital databases of the single referring centers. Accuracy of previous MDRO events and steroids and antimicrobial drug treatments might be limited, although these factors play a major role in routine management of ICU patients, and we do not expect major gaps in data acquisition.

In conclusion, our in-depth analysis of incidence measures of HAIs and MDRO events contributes to increase knowledge of MDRO colonization and infections in ICU COVID-19 patients. These findings should be a priority in contributing toward IPC and antimicrobial stewardship policies for ensuring the best clinical care.

Appendix Additional information on multidrug-resistant bacteria colonization and infections in large retrospective cohort of COVID-19 mechanically ventilated patients, Milan, Italy.
