# Supplementary material for: Multidrug-Resistant Bacterial Colonization and Infections in Large Retrospective Cohort of Mechanically Ventilated COVID-19 Patients
Source: Emerg Infect Dis. 2023 Aug;29(8):1598–607. doi: 10.3201/eid2908.230115 (PMC10370845; doi:10.3201/eid2908.230115)
Supplement: Appendix — Additional information on multidrug-resistant bacteria colonization and infections in large retrospective cohort of COVID-19 mechanically ventilated patients, Milan, Italy. [file 23-0115-Techapp-s1.pdf]

Article DOI: https://doi.org/10.3201/eid2908.230115

EID cannot ensure accessibility for Supplemental Materials supplied by authors. Readers who have difficulty accessing supplementary content should contact the authors for assistance.

Multidrug-Resistant Bacterial Colonization and Infections in Large Retrospective Cohort of Mechanically Ventilated COVID-19 Patients

Appendix

Methods

Complete list of data collected and criteria for data classification

Demographic data

- Age
- Gender
- Weight, ideal weight
- Height
- Body mass index (BMI)
- Comorbidities/organ failure: Charlson Comorbidity Index, cardiac disease, lung disease, renal disease, hypertension, diabetes mellitus, smoking history, immunological deficit (at least 1 of: solid organ transplantation, bone marrow transplantation, active neoplastic disease, hematological tumors, rheumatological diseases, acquired immunodeficiency syndrome (AIDS), asplenia, chemotherapy within the previous 3 months, neutropenia (<500/microL), use of biologic drugs, use of corticosteroids (>10 mg/day prednisone or equivalent within the previous 3 months), other forms of immunosuppression including congenital/genetic immunodeficits)

Clinical Data before ICU Admittance

- date of COVID-19 symptoms onset

• date of hospital admittance

• date of ICU admittance

• antivirals (remdesivir) administration before ICU admittance

• antibiotics administration before ICU admittance: days of antibiotic intake, antibiotic class (betalactam/betalactamases inhibitors, oxacillin/cefazolin, 3–4th generation cephalosporins, 5th generation cephalosporins, cefiderocol, cerbapenems, macrolides, vancomycin/teicoplanin, daptomycin, linezolid, fluoroquinolones, aminoglycosides, colistin, fosfomycin, azoles, echinocandines, others)

• steroids administration before ICU admittance: days of steroids intake, steroids class (STANDARD prednisone or dexamethasone 6mg/day, HIGH DOSE methylprednisolone ≥1 mg/kg/day)

• MDROs colonization before ICU admittance: *E. coli* ESBL+, Klebsiella spp. ESBL+, Klebsiella spp. CARBA-R, P. aeruginosa CARBA R, Acinetobacter spp. CARBA-R, methicillin-resistant Staphylococcus aureus, Vancomycin-resistant Enterococcus faecium, others (any bacteria with resistance to at least 1 molecule in 3 or more antibiotic classes)

• MDRO infections before ICU Admittance

Clinical and laboratory data during ICU stay

• Date of ICU admittance

• Hospital of provenance

• Setting of provenance: Emergency room (ER) if ICU admission occurred within 48 hours from hospitalization; non-intensive hospital wards if ICU admission occurred after 48 hours from hospitalization; ICU if patients stayed in ICU for over 24 hours before ICU transferral.

• ICU module

• PaO2:FiO2 ratio at ICU admission

• SOFA score at ICU admission

• SAPS II score at ICU admission

• Date of mechanical ventilation start

• Date of mechanical ventilation end

Microbiological information during ICU stay

• Date of microbiological sample, type of microbiological sample

Microbiological sample	Classification_1	Classification_2
Sputum	ETA	respiratory
endotracheal aspirate	ETA	respiratory
endotracheal tube	ETA	respiratory
bronchoalveolar lavage	BAL	respiratory
right bronchoalveolar lavage	BAL	respiratory
left bronchoalveolar lavage	BAL	respiratory
pleural fluid	pleural fluid	respiratory
blood from venous catheter	central line	blood
blood from venous catheter (dialysis)	central line	blood
blood from arterial catheter	Peripheral line	blood
blood from peripheral vein	Peripheral line	blood
vascular catheter	catheter	catheter
arterial catheter	catheter	catheter
central venous catheter	catheter	catheter
central venous catheter (dialysis)	catheter	catheter
Urine	urine	urine
urine from urinary catheter	urine	urine
midstream urine sample	urine	urine
skin swab	surveillance	surveillance
pharyngeal swab	surveillance	surveillance
nasal swab	surveillance	surveillance
perianal swab	surveillance	surveillance
rectal swab	surveillance	surveillance
rectal/perianal swab	surveillance	surveillance
axillary swab	surveillance	surveillance
inguinal swab	surveillance	surveillance
fecal sample	other	other
abdominal drainage	other	other
thoracic drainage	other	other
Liquor	other	other
purulent material	other	other
vaginal secretion	other	other
wound swab	other	other
foreskin swab	other	other
tracheostomy swab	other	other
ulceral swab	other	other
eschar swab	other	other
labial swab	other	other

• Interpretation of resistance pattern of the identified microorganism (see below "MDR DEFINITIONS)

• Interpretation of microbiological sample according to attending physician: infection/colonization/contamination

• Interpretation of microbiological sample according to literature: infection/colonization/contamination. Infections were defined by the presence of significant bacterial load associated with clinical manifestations within the infection window period

(IWP,±3 days from specimen collection) (see [reference #22]: CDC. National Healthcare Safety Network (NHSN) Patient Safety Component Manual. January 2021, [reference #23]: European Centre for Disease Prevention and Control. Surveillance of healthcare-associated infections and prevention indicators in European intensive care units. Stockholm: ECDC; 2017). Isolates were classified as colonization when no adverse clinical signs or symptoms was documented. Isolates that did not meet the criteria of infection/colonization and were listed in the CDC-NHSN list of common commensals were interpreted as contaminants (Centers for Disease Control and Prevention (CDC). CDC/NHSN Common Commensals List [Internet]. 2021. Available from: https://www.cdc.gov/nhsn/xls/master-organism-com-commensals-lists.xlsx).

• For infections, interpretation of microbiological sample as new or persistent infection: the combination of a) new signs and symptoms and b) radiographic evidence (for pneumonia) or other diagnostic testing were required to consider an infection as a new infection episode (see [reference #23]: European Centre for Disease Prevention and Control. Surveillance of healthcare-associated infections and prevention indicators in European intensive care units. Stockholm: ECDC; 2017)

• For bloodstream infections, interpretation of the BSI episode as primary, secondary to another source of infection or catheter-related (see below "DIAGNOSTIC CRITERIA FOR INFECTIONS")

• For infections, interpretation of the severity of the episode as infection without sepsis, sepsis or septic shock based on clinical manifestations occurred during the infection window period (±3 days from specimen collection) (see [reference #24]: Singer M, Deutschman CS, Seymour CW, et al. The Third International Consensus Definitions for Sepsis and Septic Shock (Sepsis-3). JAMA. 2016;315(8):801–810. doi:10.1001/jama.2016.0287)

Therapeutic information during ICU stay

• Antibiotic therapy: date of start, date of end, date of change of posology, type of antibiotic therapy

Antibiotic (active ingredient)	Classification_1	Classification_2
amphotericin b	antifungal	AMB
liposomal amphotericin b	antifungal	AMB
Amikacin	no anaerobic activity	aminoglycosides
amoxicillin/ac. Clavulanic	anti-anaerobic activity	BL/BLIs
Ampicillin	anti-anaerobic activity	penicillins
Ampicillin/sulbactam	anti-anaerobic activity	BL/BLIs
Anidulafungin	antifungal	echinocandins

Azithromycin	no anaerobic activity	MLs
Caspofungin	antifungal	echinocandins
Cefazoline	no anaerobic activity	anti-Staph BLs
Cefepime	no anaerobic activity	3–4G cephalosporins
Cefiderocol	no anaerobic activity	novel anti G- cephalosporins
Cefotaxime	no anaerobic activity	3–4G cephalosporins
Ceftaroline	no anaerobic activity	novel anti G+ cephalosporins
Ceftazidime	no anaerobic activity	3–4G cephalosporins
ceftazidime/avibactam	no anaerobic activity	novel anti G- cephalosporins
ceftolozane/tazobactam	no anaerobic activity	novel anti G- cephalosporins
Ceftriaxone	no anaerobic activity	3–4G cephalosporins
Ciprofloxacin	no anaerobic activity	FQs
Clindamycin	anti-anaerobic activity	lincosamides
Colistin	no anaerobic activity	polymixins
Daptomycin	anti-anaerobic activity	glyco/lipopeptides
Fidaxomicin	other	other
Fluconazole	antifungal	azoles
Fosfomycin	no anaerobic activity	FOF
Gentamycin	no anaerobic activity	aminoglycosides
Imipenem	anti-anaerobic activity	carbapenems
isavuconazole	antifungal	azoles
Levofloxacin	no anaerobic activity	FQs
Linezolid	anti-anaerobic activity	oxazolidinones
Meropenem	anti-anaerobic activity	carbapenems
meropenem/vaborbactam	anti-anaerobic activity	novel carbapenems
metronidazole	anti-anaerobic activity	MTZ
Oxacillin	no anaerobic activity	anti-Staph BLs
penicillin G	anti-anaerobic activity	penicillins
Piperacillin	anti-anaerobic activity	penicillins
piperacillin/tazobactam	anti-anaerobic activity	BL/BLIs
Rifampin	no anaerobic activity	RIF
Rifaximin	other	other
trimethoprim/sulfamethoxazole	no anaerobic activity	SXT
Tigecycline	anti-anaerobic activity	tetracyclines
Tobramycin	no anaerobic activity	aminoglycosides
Vancomycin	anti-anaerobic activity	glyco/lipopeptides
Voriconazole	antifungal	azoles

- Steroid therapy: date of start, date of end, type of steroid therapy

Corticosteroid (active ingredient and posology)	Classification
Dexamethasone (any dosage)	STANDARD dose
Methylprednisolone <1mg/kg/die	STANDARD dose
Methylprednisolone ≥1mg/kg/die	HIGH dose

Outcome data

- Length of ICU stay
- Vital status at ICU discharge (alive/dead)

MDR definitions

Source	Definition criteria
Magiorakos et al, CMI 2012 [reference #25]	• MDR: non-susceptible to > = 1 agent in > = 3 antimicrobial categories + MRSA<br>• XDR: non-susceptible to > = 1 agent in all but < = 2 categories<br>• PDR: non-susceptible to all antimicrobial agents listed
Grasselli et al, CHEST 2021 [reference #4]	resistance to > = 1 agent in > = 3 antimicrobial categories + methicillin-resistant *Staphylococcus* spp, vancomycin-resistant *Enterococcus* spp, ESBL/AmpC/carbapenemases-producing *Enterobacterales*, carbapenem resistant gram-negative bacteria

Diagnostic criteria for infections

Microbiologically defined infections (MDI) only

Infection	Site of Culture	Bacterial Load	Clinical Signs	Also
Primary Blood Stream Infection	2 percutaneous blood samples<br>+<br>eventual blood from catheters	—	Fever/chills/hypotension<br>+<br>No further sign of localized infection	No differential time to positivity between percutaneous and catheters
	If Common Commensal organisms (i.e., diphtheroids (*Corynebacterium* spp. not *C. diphtheria*), *Bacillus* spp. (not *B. anthracis*), *Propionibacterium* spp., coagulase-negative staphylococci (including *S. epidermidis*), viridans group streptococci, *Aerococcus* spp. *Micrococcus* spp. and *Rhodococcus* spp.): necessary two or more blood specimens drawn on separate occasions			
Central line associated Blood Stream Infection[1]	percutaneous blood samples<br>+<br>catheter blood<br>or<br>catheter tip	—	Fever/chills/hypotension<br>+<br>No further sign of localized infection. Eventual erythema, swelling, purulent drainage from catheter insertion-site.	Differential time to positivity >2 h<br>or<br>positive catheter tip
	If Common Commensal organisms (i.e., diphtheroids (*Corynebacterium* spp. not *C. diphtheria*), *Bacillus* spp. (not *B. anthracis*), *Propionibacterium* spp., coagulase-negative staphylococci (including *S. epidermidis*), viridans group streptococci, *Aerococcus* spp. *Micrococcus* spp. and *Rhodococcus* spp.): necessary two or more blood specimens drawn on separate occasions			
Ventilator-associated lower respiratory tract infections[2]	Bronchoalveolar lavage	$\geq 10^4$ CFU/mL	1 of: fever, leukocytosis/leucopenia<br>+<br>1 of: worsening oxygenation, purulent secretions<br>+<br>New/progressive radiographic infiltrate (if available)	
	Endotracheal Aspirate	$\geq 10^5$ CFU/mL		
	secondary Blood Stream Infection[3]			
	Excluded organisms: "Normal respiratory flora," "normal oral flora," "mixed respiratory flora," and, unless identified from lung tissue or pleural fluid (with specimen obtained during thoracentesis or initial placement of chest tube and NOT from an indwelling chest tube), *Candida* spp, coagulase-negative staphylococci, *Enterococcus* spp			
Catheter-associated Urinary Tract Infection[4]	urine culture[5]	$\geq 10^5$ CFU/mL	Fever/chills/hypotension<br>+<br>No further sign of localized infection	
	secondary Blood Stream Infection[3]			
	Excluded organisms: "mixed flora," *Candida* spp, yeast, mold, dimorphic fungi, parasites			
Clostridioides difficile Colitis	Unformed stool culture	—	Fever/chills/hypotension<br>+<br>Unformed stool	Enzyme immunoassay positive for C. difficile GDH + toxins A/B or positive NAAT
COVID-Associated Pulmonary Aspergillosis	Proven<br>Histopathological or direct microscopic detection of fungal hyphae, showing invasive growth with associated tissue damage<br>or<br>Aspergillus recovered by culture or microscopy or histology or PCR obtained by a sterile aspiration or biopsy from a pulmonary site, showing an infectious disease process			
	Probable – tracheobronchitis<br>Tracheobronchial ulceration, nodule, pseudomembrane, plaque, or eschar seen on bronchoscopic analysis<br>+<br>One of the following: microscopic detection of fungal elements in bronchoalveolar lavage indicating a mold; positive bronchoalveolar lavage culture or PCR; serum galactomannan index >0.5; bronchoalveolar lavage galactomannan index ≥1.0			
	Probable – other pulmonary forms<br>Pulmonary infiltrate or cavitating infiltrate, preferably documented by chest CT and not attributed to another cause<br>+<br>One of the following: microscopic detection of fungal elements in bronchoalveolar lavage indicating a mold; positive bronchoalveolar lavage culture or PCR; serum galactomannan index >0.5; bronchoalveolar lavage galactomannan index ≥1.0			
	Possible - other pulmonary forms<br>Pulmonary infiltrate or cavitating infiltrate, preferably documented by chest CT and not attributed to another cause<br>+<br>One of the following: microscopic detection of fungal elements in non-bronchoalveolar lavage indicating a mold; positive non-bronchoalveolar lavage culture; single non-bronchoscopic lavage galactomannan index >4.5; non-bronchoscopic lavage galactomannan index >1.2 twice or more; non-bronchoscopic lavage galactomannan index >1.2 plus positive PCR			

Infection	Site of Culture	Bacterial Load	Clinical Signs	Also
Candidemia/ Invasive Candidiasis	Proven<br>*Candida* spp. identified from one or more blood specimens obtained by culture or non-culture microbiologic testing methods			
	Presumptive<br>Fever/chills/hypotension + risk factors (i.e., Candida score, Candida Colonization Index) + positive fungal biomarkers (i.e., 1,3-β-d-glucan BDG) + exclusion of alternative diagnoses			
CFU, Colony forming units. [1] at least 48 h after catheter positioning. Central line colonization: positive catheter blood or catheter tip and negative percutaneous blood samples. [2] at least 48 h after intubation. [3] positive blood specimen containing at least one eligible matching organism to the site-specific specimen o meeting the site-specific infection criteria. [4] at least 48 h after indwelling urinary catheter positioning. All the patients had urinary indwelling catheters. [5] if urinary catheter in place for more than 5 d, the catheter is removed, a new catheter is repositioned and a second specimen is collected.				

Statistical Analysis

Patients' characteristics were described overall and for selected groups of interest such ass MDROs acquired before/after ICU admittance, MDROs infection/colonization. Median (interquartile range, IQR) are reported for continuous variables, and number (percentages) for categorical variables. Groups were compared with parametric or nonparametric tests, according to data distribution, for continuous variables and with Pearson Chi-square test (or Fisher exact test when appropriate) for categorical variables. Crude incidence rates per 1000 patient-days (IR/$1000_{\text{patient-days}}$) and relative 95% confidence intervals (95%CIs) were calculated considering for each patient any first species-specific MDRO colonization and/or each new MDRO/non-MDRO HAI. Since we speculate that some patients have greater propensities for recurrent events than others, and thus events within a single patient may not be considered as independent observations, we calculated incidence rates considering the negative binomial distribution, as already proposed (see [reference #26]: Glynn RJ, Buring JE. Ways of measuring rates of recurrent events. BMJ Br Med J [Internet]. 1996 Feb 2 [cited 2022 Oct 20];312(7027):364. Available from: /pmc/articles/PMC2350293/?report = abstract) The time considered for IRs estimates was set from ICU admission to discharge, except for VALRTI where total intubation time was considered. To measure agreement between different methods for classifying microbiological isolates as infections, Cohen's kappa coefficient (κ) was applied. All tests were two-sided, and $p < 0.05$ was chosen to indicate statistical significance. Software SAS 9.4 (SAS Institute) was used for statistical analysis.

Literature Review

To identify relevant studies on MDRO events in COVID-19 ICU patients indexed on PubMed and/or Embase, we used the following string: ((“COVID-19”[MeSH Major Topic]) OR (COVID)) AND (“ICU”[Title/Abstract] OR “INTENSIVE CARE”[Title/Abstract] OR “CRITICAL*”[Title/Abstract]) AND (“MDR”[Title/Abstract] OR “multidrug-

resist*"[Title/Abstract] OR "multidrug resist*"[Title/Abstract] OR "carbapenem-resistant"[Title/Abstract]) AND ((COLONIZATION) OR (INFECTION) OR (EPIDEMIOLOGY)). The review was conducted based on the PRISMA guidelines for reviews (Appendix Figure 3). The last search was performed on September 19, 2022.

References

1. Centers for Disease Control and Prevention. National Healthcare Safety Network (NHSN) Patient Safety Component Manual. January 202 [cited 2023 Jun 6]. https://www.cdc.gov/nhsn/pdfs/pscmanual/pcsmanual_current.pdf

2. Koehler P, Bassetti M, Chakrabarti A, Chen SC, Colombo AL, Hoenigl M, et al.; European Confederation of Medical Mycology; International Society for Human Animal Mycology; Asia Fungal Working Group; INFOCUS LATAM/ISHAM Working Group; ISHAM Pan Africa Mycology Working Group; European Society for Clinical Microbiology; Infectious Diseases Fungal Infection Study Group; ESCMID Study Group for Infections in Critically Ill Patients; Interregional Association of Clinical Microbiology and Antimicrobial Chemotherapy; Medical Mycology Society of Nigeria; Medical Mycology Society of China Medicine Education Association; Infectious Diseases Working Party of the German Society for Haematology and Medical Oncology; Association of Medical Microbiology; Infectious Disease Canada. Defining and managing COVID-19-associated pulmonary aspergillosis: the 2020 ECMM/ISHAM consensus criteria for research and clinical guidance. Lancet Infect Dis. 2021;21:e149–62. PubMed https://doi.org/10.1016/S1473-3099(20)30847-1

Appendix Table 1. Characteristics of the 435 patients admitted to ICU, overall and for patients with and without MDRO isolates in the first 48 h (irrespective of MDRO developing (or not) during ICU stay)

PATIENTS CHARACTERISTICS	Total N = 435	$MDR_{≤48h}$ N = 88	No-MDR or $MDR_{>48h}$ N = 347
Age, years	65.0 (59.0–71.0)	65.0 (58.0–70.5)	65.0 (59.0–71.0)
Gender, female	117 (26.9)	22 (25.0)	95 (27.4)
BMI, kg/m2	28.0 (26.0–31.0)	28.0 (26.0–31.0)	28.0 (26.0–31.0)
Obesity (BMI >30)	284 (65.3)	59 (67.1)	225 (64.8)
Ever Smoker	87 (20.0)	21 (23.9)	66 (19.0)
Comorbidities			
Hypertension	225 (51.7)	44 (50.0)	181 (52.2)
Cardiovascular disease	115 (26.5)	23 (26.1)	92 (26.6)
Pneumopathy	62 (14.3)	14 (15.9)	48 (13.8)
Neuropathy	19 (4.4)	5 (5.7)	14 (4.0)
Diabetes	92 (21.2)	23 (26.1)	69 (19.9)
Immunological deficits*	29 (6.7)	7 (8.0)	22 (6.3)
Total no. of comorbidities			
0	83 (19.1)	17 (19.3)	66 (19.0)
1	141 (32.4)	26 (29.6)	115 (33.1)
2	109 (25.1)	22)25.0)	87 (25.1)

PATIENTS CHARACTERISTICS	Total<br>N = 435	$MDR_{\le 48h}$<br>N = 88	No-MDR or $MDR_{>48h}$<br>N = 347
>3	102 (23.9)	23 (26.1)	79 (22.8)
Setting characteristics	Total, n = 435	$MDR_{\le 48h}$, n = 88	No-MDR or $MDR_{>48h}$, n = 347
Month of ICU_{FIERA} admission			
Oct 2020	20 (4.6)	2 (2.3)	18 (5.2)
Nov 2020	97 (22.3)	21 (23.9)	76. (21.9)
Dec 2020	53 (12.2)	19 (21.6)	34 (9.8)
Jan 2021	56 (12.9)	8 (9.1)	43 (13.8)
Feb 2021	53 (12.2)	12 (13.6)	41 (11.8)
Mar 2021	60 (13.8)	6 (6.8)	54 (15.6)
Apr 2021	17 (3.9)	5 (5.7)	12 (3.5)
Setting of provenance [α]			
ER	117 (26.9)	31 (35.2)	86 (24.8)
Non-intensive hospital wards	117 (26.9)	15 (17.1)	102 (29.4)
ICU	201 (46.2)	42 (47.7)	159 (45.8)
Center A	53 (12.2)	6 (6.8)	47 (13.5)
Center B	37 (8.5)	9 (10.2)	28 (8.1)
Center C	37 (8.5)	7 (8.0)	30 (8.7)
Center D	37 (8.5)	14 (15.9)	23 (6.6)
Center E	33 (7.6)	6 (6.8)	27 (7.8)
Center F	22 (5.1)	0 (0)	22 (6.3)
Center G	17 (3.9)	6 (6.8)	11 (3.2)
Center H	17 (3.9)	1 (1.1)	16 (4.6)
Center I	16 (3.7)	3 (3.4)	13 (13.8)
Center J	15 (3.6)	2 (2.3)	13 (13.8)
Center K	12 (2.8)	4 (4.6)	8 (2.3)
Other 36 centers with <10 patients	139 (32.0)	30 (34.1)	109 (31.4)
DISEASE CHARACTERISTICS PRIOR TO ICU ADMISSION			
time between first symptoms and hospitalization, days	5.0 (3.0–7.0)	6.0 (3.0–7.0)	5.0 (3.0–7.0)
time between hospitalization and ICU admission, days [β]	5.0 (2.0–9.0)	6.0 (3.0–12.0)	5.0 (2.0–8.0)
time between hospitalization and MV start, days	3.0 (1.0–6.5)	4.0 (2.0–7.0)	3.0 (1.0–6.0)
Steroid therapy, standard dose [#]	282 (64.8)	54 (61.4)	228 (65.7)
Steroid therapy, high dose [# γ]	56 (13.0)	17 (19.8)	39 (11.3)
Antibiotic therapy [δ]			
None	151 (34.7)	25 (28.4)	126 (36.3)
1 class	154 (35.4)	29 (33.0)	125 (36.0)
2 classes	95 (21.8)	22 (25.0)	73 (21.0)
≥3 classes	35 (8.1)	12 (13.6)	23 (6.6)
MDROs infection/colonization	12 (2.8)	8 (9.1)	4 (1.2)
PaO2 to FIO2 ratio at ICU admission, mmHg	134.0 (105.0–180.0)	126.0 (100.5–179.0)	137.0 (106.0–180.0)
200	74 (17.0)	16 (18.2)	58 (16.7)
<100 and ≥200	268 (61.6)	50 (56.8)	50 (56.8)
≤100	93 (21.4)	22 (25.0)	71 (20.5)
OUTCOME			
Alive at discharge	286 (65.8)	57 (64.8)	229 (66.0)
Deceased	149 (34.3)	31 (35.2)	118 (34.0)
Length of MV, days	17.0 (11.0–28.0)	19.0 (13.0–33.0)	16.0 (10.0–26.0)
ICU stay, days	20.0 (12.0–32.0)	17.0 (11.0–29.0)	21.0 (13.0–33.0)

Categorical variables are expressed as frequency (percentages), continuous variables are expressed as median (interquartile range). [α] Chi-square test p-value = 0.032; [β] Mann–Whitney U test p-value = 0.006; [γ] Chi-square test p-value = 0.036; [δ] Chi-square for trend p-value = 0.021.
Legend: BMI body mass index, ER emergency room, ICU intensive care unit, MV mechanical ventilation, MDROs multidrug resistant organisms; * at least 1 of: solid organ transplantation, active neoplastic disease, hematological disease, rheumatological disease, AIDS, asplenia, chemotherapy in the past 3 mo, neutropenia (N < 500 / microL), use of biologics, use of corticosteroids (>10 mg / day prednisone or equivalent>3 mo pre-hospitalization), other forms of immunosuppression (including congenital / genetic forms); [#] standard dose in case of use of dexamethasone or methylprednisolone <1 mg / kg / day, high dose in case of use of methylprednisolone greater than or equal to 1 mg / kg / day or equivalent (patients could have received both standard and high dose of steroid.

Appendix Table 2. Duration between hospitalization and transfer to ICU based on the patients' setting of provenance

Setting of provenance	Days between hospitalization and transfer to ICU by setting of provenance	N	Median	Lower Quartile	Upper Quartile	p value*
ER	MDR = <48h	15	1.0	0.0	2.0	0.826
	MDR >48h	55	1.0	1.0	2.0	
	no-MDR	47	1.0	0.0	2.0	
non-intensive hospital wards	MDR = <48h	42	5.5	4.0	8.0	0.780
	MDR >48h	92	6.0	4.0	8.0	
	no-MDR	67	6.0	4.0	9.0	
ICU	MDR = <48h	31	11.5	8.0	18.0	0.091
	MDR >48h	60	9.0	6.0	13.0	
	no-MDR	26	7.0	4.0	12.0	

* Kruskal-Wallis test

Appendix Table 3. Details on bacterial species and time of acquisition of the subgroup of patients who developed MDRO infection from the same MDRO colonizing bacteria

Bacterial species	Days between ICU admission and colonization	Colonization sample	Days between ICU admission and Infection	Infection sample
Enterococcus faecalis	4	SURV SWAB	7	BLOOD
Enterococcus faecium	5	SURV SWAB	13	BLOOD
Enterococcus faecium	7	SURV SAWB	33	BLOOD
Enterococcus faecium	22	SURV SWAB	26	BLOOD
Escherichia coli	3	SURV SWAB	15	URINE
Klebsiella spp	6	ETA/BAL	9	ETA/BAL
Klebsiella spp	8	SURV SWAB	14	ETA/BAL
Klebsiella spp	9	ETA/BAL	16	ETA/BAL
Klebsiella spp	11	SURV SWAB	13	ETA/BAL
Klebsiella spp	11	SURV SWAB	15	ETA/BAL
Klebsiella spp	11	SURV SWAB	17	ETA/BAL
Klebsiella spp	15	SURV SWAB	27	ETA/BAL
Klebsiella spp	20	SURV SWAB	55	BLOOD
Klebsiella spp	30	SURV SWAB	33	BLOOD
Proteus mirabilis	21	ETA/BAL	24	ETA/BAL
Providencia stuartii	10	ETA/BAL	12	BLOOD
Pseudomonas aeruginosa	14	ETA/BAL	18	ETA/BAL
Pseudomonas aeruginosa	54	ETA/BAL	57	ETA/BAL
Staphylococcus aureus	3	SURV SWAB	6	ETA/BAL
Staphylococcus aureus	5	SURV SWAB	6	ETA/BAL
Staphylococcus aureus	8	SURV SWAB	9	ETA/BAL
Staphylococcus aureus	14	ETA/BAL	24	ETA/BAL
Staphylococcus aureus	25	SURV SWAB	30	ETA/BAL
Staphylococcus aureus	47	ETA/BAL	51	ETA/BAL

Appendix Table 4. Analysis of concordance in the interpretation of bacterial isolates as colonization or healthcare-associated infection (HAIs) between clinical criteria and retrospective evaluation according to international guidelines (N = 5213, isolates retrospectively classified as contaminants were excluded)

Literature criteria (retrospective evaluation)	Clinical diagnosis (real-life interpretation of microbiological reports)		
	colonization	HAI	Total
Colonization	2864 (93.9)	185 (6.1)	3049 (58.5)
HAI	66 (3.1)	2096 (96.9)	2162 (41.5)
Total	2930 (56.2)	2281 (43.8)	5211
simple kappa coefficient			
Estimate	Standard Error	95% Confidence Limits	
0.9016	0.0061	0.8897	0.9134
Numbers are expressed as frequency (percentages). Concordance between literature criteria and clinical diagnosis is reported in bold			

Appendix Table 5. MDRO and antibiotic-susceptible (non-MDRO) bacterial isolates interpreted as healthcare-associated infections (HAIs), by bacterial species and infection site

Species	Total (n = 546)	VALRTI (n = 359)		BLOOD (n = 141)		UTI (n = 40)		Other (n = 6)		Total HAIs	
		MDRO (n = 82)	non-MDRO (n = 277)	MDRO (n = 42)	non-MDRO (n = 99)	MDRO (n = 4)	non-MDRO (n = 36)	MDRO (n = 2)	non-MDRO (n = 4)	MDRO (n = 130)	non-MDRO (n = 416)
Total Gram positive	220	21	82	27	64	2	20	2	2	52	168
Total Gram negative	326	61	195	15	35	2	16	0	2	78	248
Acinetobacter baumannii	9	7	0	2	0	0	0	0	0	9	0
Bacillus clausii	1	0	0	0	1	0	0	0	0	0	1
Citrobacter spp	10	1	9	0	0	0	0	0	0	1	9
Clostridioides difficile	2	0	0	0	0	0	0	0	2	0	2
coagulase negative Staphylococcus	20	0	0	16	3	0	1	0	0	16	4
Corynebacterium spp	7	0	3	0	4	0	0	0	0	0	7
Delftia acidovorans	3	0	2	0	1	0	0	0	0	0	3
Enterobacter spp	31	1	27	0	2	0	1	0	0	1	30
Enterococcus faecalis	62	0	7	2	36	0	16	0	1	2	60
Enterococcus faecium	16	1	1	3	9	0	2	0	0	4	12
Enterococcus spp	1	0	0	0	0	1	0	0	0	1	0
Escherichia coli	26	5	14	0	1	1	5	0	0	6	20
Fusobacterium necrophorum	1	0	0	0	1	0	0	0	0	0	1
Haemophilus influenzae	3	0	3	0	0	0	0	0	0	0	3
Hafnia alvei	8	1	4	0	3	0	0	0	0	1	7
Klebsiella spp	87	23	40	10	12	1	1	0	0	34	53
Legionella pneumophila	1	0	1	0	0	0	0	0	0	0	1
Morganella morganii	5	0	5	0	0	0	0	0	0	0	5
Proteus mirabilis	9	3	5	0	1	0	0	0	0	3	6
Providencia stuartii	1	0	0	1	0	0	0	0	0	1	0
Pseudomonas aeruginosa	101	20	62	2	9	0	8	0	0	22	79
Pseudomonas spp	1	0	1	0	0	0	0	0	0	0	1
Serratia marcescens	7	0	5	0	1	0	1	0	0	0	7
Staphylococcus aureus	102	19	63	6	10	1	1	2	0	28	74
Stenotrophomonas maltophilia	21	0	17	0	4	0	0	0	0	0	21
Streptococcus agalactiae	2	1	1	0	0	0	0	0	0	1	1
Streptococcus anginosus	2	0	0	0	1	0	0	0	1	0	2
Streptococcus pneumoniae	7	0	7	0	0	0	0	0	0	0	7

Appendix Table 6. MDRO and antibiotic-susceptible (non-MDRO) bacterial isolates interpreted as healthcare-associated infections (HAIs), by bacterial species and infection severity

Species	Total (n = 546)	no sepsis (n = 266)		sepsis (n = 194)		septic shock (n = 86)		Total HAIs	
		MDRO (n = 61)	non-MDRO (n = 205)	MDRO (n = 52)	non-MDRO (n = 142)	MDRO (n = 17)	non-MDRO (n = 69)	MDRO (n = 130)	non-MDRO (n = 416)
Total Gram positive	220	21	85	25	55	6	22	52	168
Total Gram negative	326	40	120	27	87	11	47	78	248
Acinetobacter baumannii	9	3	0	2	0	4	0	9	0
Bacillus clausii	1	0	0	0	0	0	1	0	1
Citrobacter spp	10	1	4	0	5	0	0	1	9
Clostridioides difficile	2	0	1	0	0	0	1	0	2
coagulase negative Staphylococcus	20	4	2	10	1	2	1	16	4
Corynebacterium spp	7	0	1	0	5	0	1	0	7
Delftia acidovorans	3	0	2	0	0	0	1	0	3
Enterobacter spp	31	0	15	1	11	0	4	1	30
Enterococcus faecalis	62	1	29	1	22	0	9	2	60
Enterococcus faecium	16	2	6	1	5	1	1	4	12
Enterococcus spp	1	1	0	0	0	0	0	1	0
Escherichia coli	26	3	11	2	7	1	2	6	20
Fusobacterium necrophorum	1	0	0	0	1	0	0	0	1
Haemophilus influenzae	3	0	3	0	0	0	0	0	3
Hafnia alvei	8	0	3	0	3	1	1	1	7
Klebsiella spp	87	19	24	14	17	1	12	34	53
Legionella pneumophila	1	0	1	0	0	0	0	0	1
Morganella morganii	5	0	5	0	0	0	0	0	5
Proteus mirabilis	9	3	5	0	1	0	0	3	6
Providencia stuartii	1	0	0	1	0	0	0	1	0
Pseudomonas aeruginosa	101	11	33	7	35	4	11	22	79
Pseudomonas spp	1	0	1	0	0	0	0	0	1
Serratia marcescens	7	0	0	0	1	0	6	0	7
Staphylococcus aureus	102	13	39	12	20	3	15	28	74
Stenotrophomonas maltophilia	21	0	12	0	6	0	3	0	21
Streptococcus agalactiae	2	0	1	1	0	0	0	1	1
Streptococcus anginosus	2	0	1	0	1	0	0	0	2
Streptococcus pneumoniae	7	0	6	0	1	0	0	0	7

Appendix Table 7. Steroid administration before MDROs events. Both administration before and during ICU stay are considered. Comparison between no-MDR, $MDR_{COL>48h}$ and $MDR_{INF>48h}$ groups. $MDR_{INF>48h}$ patients are divided in subgroups based on the diagnosis of prior MDRO colonization.

	Steroid administration before MDRO occurrence*		
	YES	NO	Total
no-MDR	132 (94.3)	8 (5.7)	140 (41.4)
$MDR_{COL>48h}$	98 (95.1)	5 (4.9)	103 (30.5)
$MDRO_{INF>48h}$	83 (87.4)	12 (12.6)	95 (28.1)
MDRO Infection only**	40 (85.1)	7 (14.9)	47 (13.9)
MDR colonization and subsequent MDRO infection (different species)	19 (90.5)	2 (9.5)	21 (6.2)
MDR colonization and subsequent MDRO infection (same species)	24 (88.9)	3 (11.1)	27 (8.0)
Total	251 (74.3)	87 (25.7)	338
Numbers are expressed as frequency (percentages). * administration during ICU stay for no-MDR patients; ** 23 patients with subsequent MDRO colonization by different species. Nine patients are not reported for missing information on steroid therapy			

Appendix Table 8. Antibiotic administration before MDROs events, comparison between no-MDR and $MDR_{>48h}$ groups. To balance intakes between groups, only administration occurred during the first 10 d of ICU is considered (which represents the upper quartile (Q3) of the time from ICU admission to first MDROs isolation).

Patients group	Antibiotic administration before MDRO occurrence or within 10 d from ICU admission, whichever comes first*		
	YES	NO	Total
no-MDR	18 (12.9)	122 (87.1)	140 (41.5)
$MDR_{>48h}$	116 (58.9)	81 (41.1)	197 (58.5)
Total	134 (39.8)	203 (60.2)	337
Numbers are expressed as frequency (percentages). * chi-square test p-value <0.001. Ten patients are not reported for missing information on antibiotic therapy			

	mean (95% CI)	median (Q1-Q3)
Length of ICU stay, days<br>• no-MDR<br>• $MDR_{>48h}$	20.5 (17.8–23.3)<br>28.5 (26.1–30.8)	15.5 (10–24)<br>25 (16–37)
Days from ICU admission to MDROs event<br>• no-MDR<br>• $MDR_{>48h}$	-<br>8.2 (7.1–9.2)	-<br>7 (4–10)

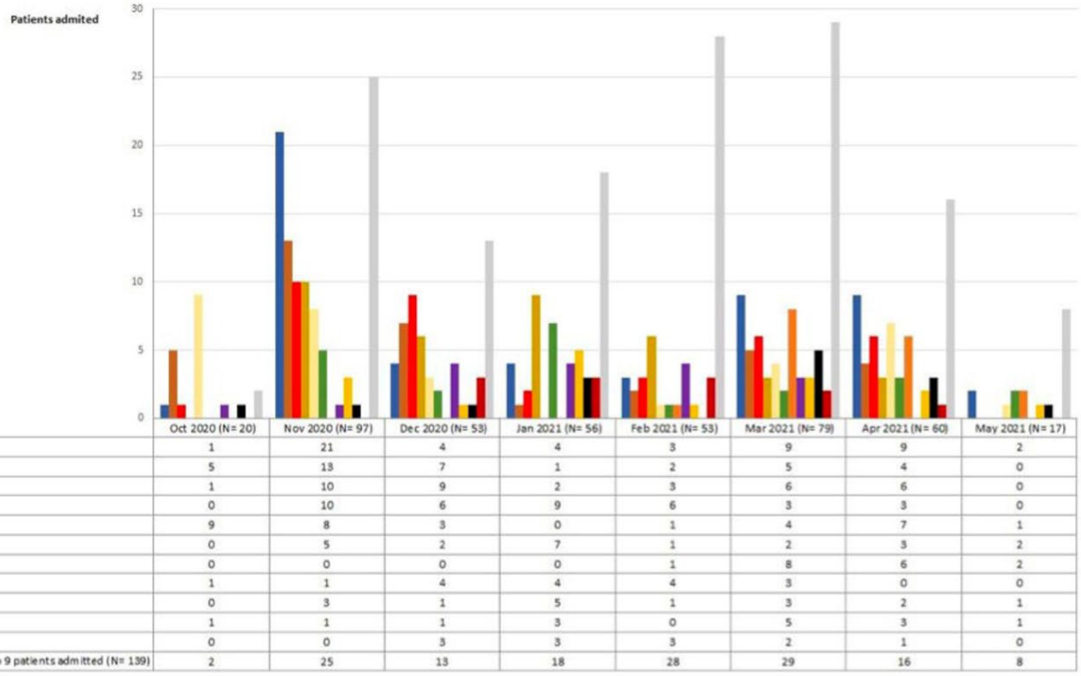

	Oct 2020 (N= 20)	Nov 2020 (N= 97)	Dec 2020 (N= 53)	Jan 2021 (N= 56)	Feb 2021 (N= 53)	Mar 2021 (N= 79)	Apr 2021 (N= 60)	May 2021 (N= 17)
Center A (N= 53)	1	21	4	4	3	9	9	2
Center B (N= 37)	5	13	7	1	2	5	4	0
Center C (N= 37)	1	10	9	2	3	6	6	0
Center D (N= 37)	0	10	6	9	6	3	3	0
Center E (N= 33)	9	8	3	0	1	4	7	1
Center F (N= 22)	0	5	2	7	1	2	3	2
Center G (N= 17)	0	0	0	0	1	8	6	2
Center H (N= 17)	1	1	4	4	4	3	0	0
Center I (N= 16)	0	3	1	5	1	3	2	1
Center J (N= 15)	1	1	1	3	0	5	3	1
Center K (N= 12)	0	0	3	3	3	2	1	0
Other 36 centers with 1 to 9 patients admitted (N= 139)	2	25	13	18	28	29	16	8

Appendix Figure 1. Trend of patient enrollment by referring hospital per month.

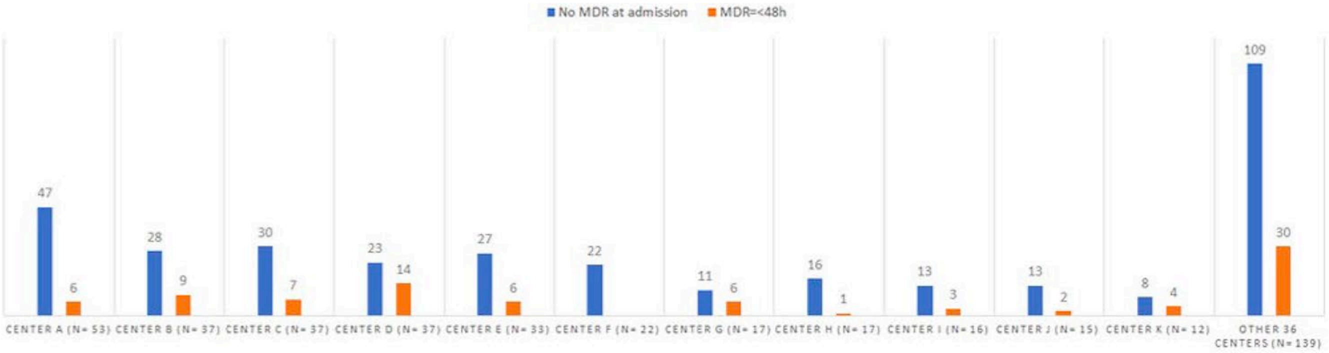

Appendix Figure 2. Number of patients with MDRO isolated in the first 48 hours from admission by referring hospital.

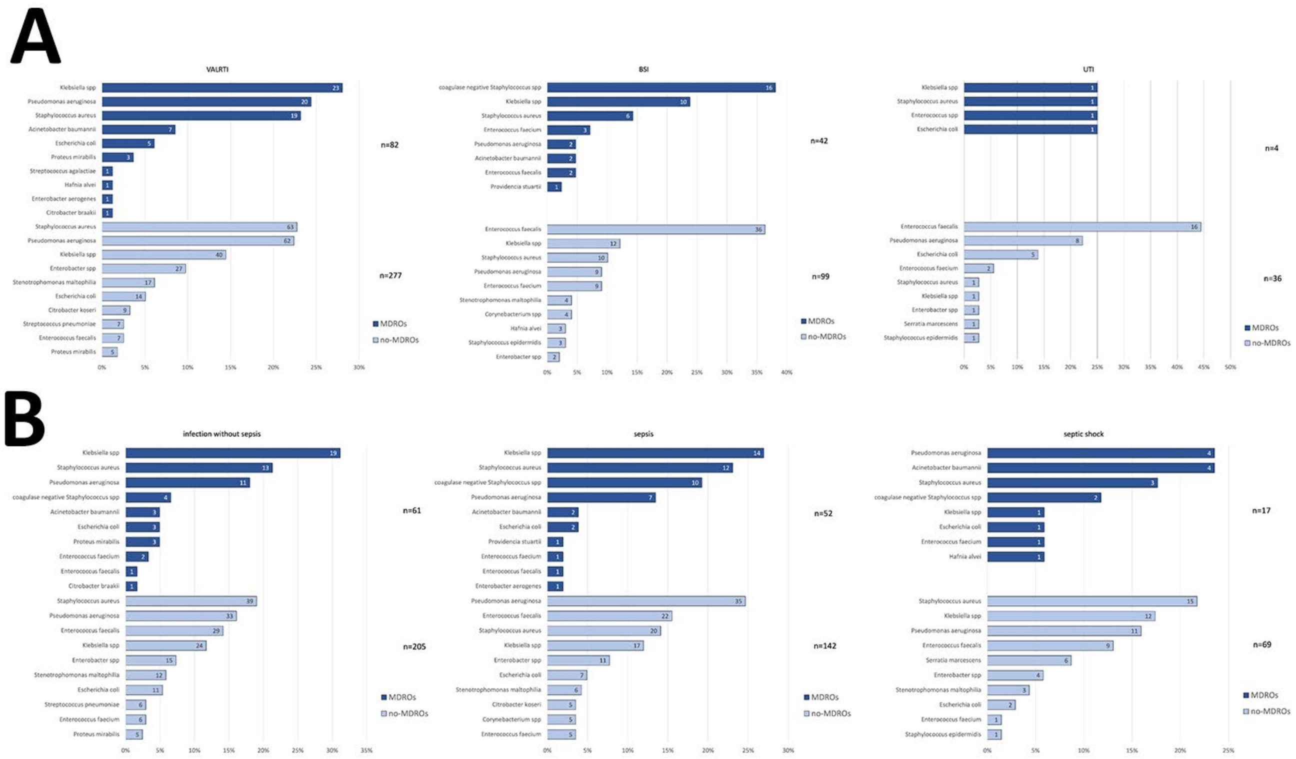

Appendix Figure 3. Multidrug-resistant bacterial colonization and infections in large retrospective cohort of COVID-19 mechanically ventilated patients. Etiology of microbiologically confirmed infections according to infection site (A) and disease severity (B).The 10 most frequent pathogens are reported as a percentage of all positive samples of that type. The number at the end of each bar indicates the total number of positive samples for the pathogen. Numbers annotated on the plots indicate the total number of organisms for each subgroup. Pathogen identification is stratified into MDRO (blue) and non-MDRO (light blue) isolates. Details with absolute numbers for each bacterial species are reported in Appendix Tables 5, 6 (https://wwwnc.cdc.gov/EID/article/29/8/23-0115-App1.pdf). MDRO, multidrug-resistant organism

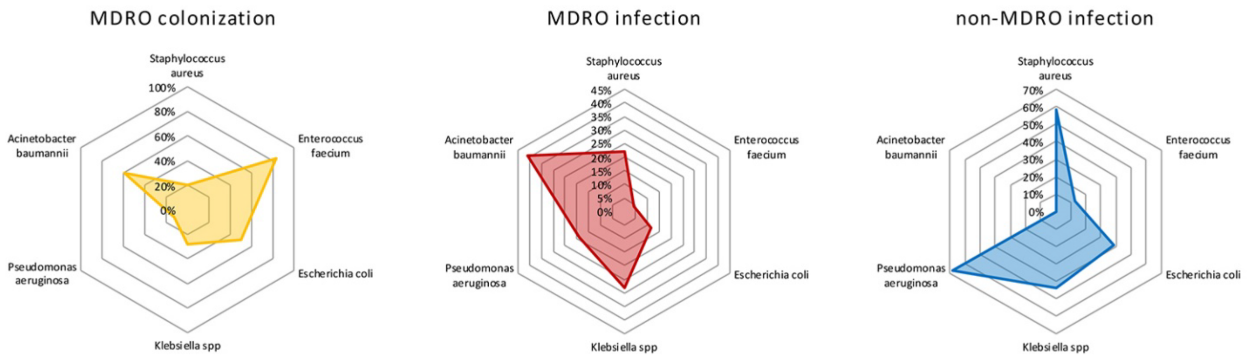

bacterial species	n° total isolates	n° MDRO colonization	% MDRO colonization	n° MDRO infection	% MDRO infection	n° non-MDRO infection	% non-MDRO infection
Staphylococcus aureus	127	25	20%	28	22%	74	58%
Enterococcus faecium	96	80	83%	4	4%	12	13%
Escherichia coli	52	26	50%	6	12%	20	38%
Klebsiella spp	121	34	28%	34	28%	53	44%
Pseudomonas aeruginosa	116	15	13%	22	19%	79	68%
Acinetobacter baumannii	22	13	59%	9	41%	0	0%

Appendix Figure 4. Radar chart and table of the most frequently isolated bacterial species. Proportions within species of MDRO colonization, MDRO infection and non-MDRO infection among the most frequently isolated bacteria of the WHO priority pathogens list are reported.
